# Host genetics, phenotype and geography structure the microbiome of a foundational seaweed

**DOI:** 10.1111/mec.16378

**Published:** 2022-02-19

**Authors:** Georgina Wood, Peter D. Steinberg, Alexandra H. Campbell, Adriana Vergés, Melinda A. Coleman, Ezequiel M. Marzinelli

**Affiliations:** ^1^ 54761 School of Life and Environmental Sciences The University of Sydney Sydney New South Wales Australia; ^2^ 54761 Centre for Marine Science and Innovation School of Biological, Earth and Environmental Sciences UNSW Sydney Sydney New South Wales Australia; ^3^ 54761 Sydney Institute of Marine Science Sydney New South Wales Australia; ^4^ 54761 Singapore Centre for Environmental Life Sciences Engineering Nanyang Technological University Singapore Singapore; ^5^ 5333 USC Seaweed Research Group University of the Sunshine Coast Sunshine Coast Queensland Australia; ^6^ Department of Primary Industries National Marine Science Centre Coffs Harbour New South Wales Australia

**Keywords:** genetics, holobiont, kelp, macroalga, marine ecology, phenotype

## Abstract

Interactions between hosts and their microbiota are vital to the functioning and resilience of macro‐organisms. Critically, for hosts that play foundational roles in communities, understanding what drives host–microbiota interactions is essential for informing ecosystem restoration and conservation. We investigated the relative influence of host traits and the surrounding environment on microbial communities associated with the foundational seaweed *Phyllospora comosa*. We quantified 16 morphological and functional phenotypic traits, including host genetics (using 354 single nucleotide polymorphisms) and surface‐associated microbial communities (using 16S rRNA gene amplicon sequencing) from 160 individuals sampled from eight sites spanning *Phyllospora's* entire latitudinal distribution (1,300 km). Combined, these factors explained 54% of the overall variation in *Phyllospora's* associated microbial community structure, much of which was related to the local environment (~32%). We found that putative “core” microbial taxa (i.e., present on all *Phyllospora* individuals sampled) exhibited slightly higher associations with host traits when compared to “variable” taxa (not present on all individuals). We identified several key genetic loci and phenotypic traits in *Phyllospora* that were strongly related to multiple microbial amplicon sequence variants, including taxa with known associations to seaweed defence, disease and tissue degradation. This information on how host‐associated microbial communities vary with host traits and the environment enhances our current understanding of how “holobionts” (hosts plus their microbiota) are structured. Such understanding can be used to inform management strategies of these important and vulnerable habitats.

## 1 INTRODUCTION

Host‐associated microbiota play a critical role in the functioning, health, survival, resilience and adaptation of eukaryotic organisms (Egan et al., 2013; McFall‐Ngai et al., 2013; Rosenberg & Zilber‐Rosenberg, 2018). Indeed, it has been suggested that hosts and their microbiome form a coherent biological entity—or “holobiont” (Dittami et al., 2021; Margulis, 1991; Rohwer et al., 2002)—which needs to be studied holistically to better understand the ecology and evolution of eukaryotic hosts (Wilkins et al., 2019; Zilber‐Rosenberg & Rosenberg, 2008). This holistic approach can inform management interventions that confer resilience and increase the adaptive capacity of hosts (Breed et al., 2019; Coleman & Goold, 2019; van Oppen et al., 2015; Wood et al., 2019). To develop such interventions, identifying the underlying factors that structure microbial communities is an early, critical step (Trevathan‐Tackett et al., 2019).

Free‐living microbial communities are strongly driven by environmental factors such as temperature, light, the chemical environment and dispersal limitation (Gusareva et al., 2019; Hellweger et al., 2014; Rusch et al., 2007; de Vries et al., 2012). While the environment may also act as a source for, and influence on, host‐associated microbiota, microbial communities are probably also shaped by strong selective forces arising from their host (Bauer et al., 2018; Coyte et al., 2015). For example, particular taxa may be associated with or excluded from a community based on host variation in chemical composition, morphology or condition (Arumugam et al., 2011; Neefjes et al., 2011; Srinivas et al., 2013). These host characteristics may be strongly determined by underlying host genetics or host interactions with the local environment (Benson et al., 2010; Bulgarelli et al., 2013; Rawls et al., 2006).

Much of our understanding of host–microbial interactions is still largely limited to humans or other mammalian model systems (e.g., mice), economically important species such as domesticated plants and livestock (Blekhman et al., 2015; Wang et al., 2016), or other specific model systems (Bosch and Miller, 2016; McFall‐Ngai, 2013). This limits inferences and application to other systems, particularly for nonmodel organisms in their natural environment. Marine environments in particular have substantial differences from terrestrial systems that probably mean that insights from terrestrial systems do not hold. For example, marine environments are often well connected, and while they are characterized by lower environmental variability across time (i.e., are more stable during particular life stages), many species have complicated life histories spanning a range of habitats, which are characterized by vastly different physical and biogeochemical factors (Steele et al., 2019). Recently, there has been an increase in studies demonstrating the presence of large and often highly diverse communities of marine host‐associated microorganisms in foundational taxa such as seaweeds and seagrasses (e.g., Fahimipour et al., 2017; Marzinelli et al., 2015). Understanding how these host‐associated microbial communities are structured and how differences or changes in community structure can impact the host is particularly important for such foundational species, as they underpin biodiversity and ecosystem functioning, and impacts can cascade throughout an entire ecosystem.

Seaweed forests are dominant habitats that underpin coastal biodiversity and the functioning of temperate reefs (Steneck and Johnson, 2013). Despite being surrounded by the same “microbial soup” within any one location, seaweeds host diverse biofilms on their surface (Egan et al., 2008; Wahl et al., 2012) that are distinct among different species (Lachnit et al., 2009) and from the surrounding sediment or seawater (Burke et al., 2011; Roth‐Schulze et al., 2016). Seaweed‐associated microbial communities play important roles in seaweed development, reproduction, functioning and defence (see Egan et al., 2013; Hollants et al., 2013; Singh & Reddy, 2016 for reviews). They are also key players in biotransformation and nutrient cycling in the oceans due to their ability to decompose algal cell walls (Goecke et al., 2010; Hollants et al., 2013; Michel et al., 2006). Recent evidence suggests that different seaweed microbial communities can also be strongly associated with differences in host condition (Campbell et al., 2011, 2015; Marzinelli et al., 2015; Qiu et al., 2019). While it is often reported that seaweed‐associated microbial communities are structured by functional traits rather than taxonomy (Burke et al., 2011), recent work on brown and red seaweeds has shown that some seaweed hosts do indeed exert strong selectivity over their microbiota (Chen & Parfrey, 2018; Saha et al., 2020; Weigel & Pfister, 2019). The mechanisms behind this selectivity, however, remain unknown.

*Phyllospora comosa* (hereafter, *Phyllospora*) is a foundational fucoid seaweed that forms underwater forests on shallow subtidal reefs in southeastern Australia (Coleman & Wernberg, 2017). *Phyllospora* forests are ecologically and economically important, but have declined along the metropolitan coastline of Sydney, Australia's largest city (Coleman et al., 2008). *Phyllospora* is currently being restored onto Sydney's reefs in one of Australia's most ambitious marine restoration programmes (Layton et al., 2018; Vergés et al., 2020). Restoration relies on the transplantation of reproductive adults from surrounding populations (Campbell et al., 2014; Marzinelli et al., 2016). The decline of this species may have been caused by host–microbial dysbiosis, influenced by historical sewage outfall discharges (Ferrari et al., 2021). Previous sampling of surface‐associated microbial communities on transplanted *Phyllospora* showed that some components of their microbial communities remained unchanged, despite hosts being moved to a different location (Campbell et al., 2015). This suggests that associated microbial communities are at least partly influenced by underlying characteristics of the host. Improving our understanding of the mechanisms and specificity behind seaweed–microbiota interactions may aid in the development of conservation and restoration tools, similar to the bioremediation and soil inoculation tools that have been developed for agriculture and restoration on land (e.g., Bashan et al., 2014; Holguin et al., 2001; Hong & Lee, 2014).

Here, we investigated the associations of host phenotype (morphology and condition), host genetics (single nucleotide polymorphism [SNP] allele frequencies) and geography (i.e., surrounding local environment) with *Phyllospora's* surface‐associated microbial communities across its entire latitudinal distribution (12° of latitude). If surface‐associated microbial communities are driven by characteristics of the host, we predicted that *Phyllospora's* phenotypic and genetic traits would explain a greater component of the microbiota diversity, abundance and composition than geography alone. We also predicted that microbial relationships with the host would be stronger for putative “core” microbiota (i.e., microbial taxa present in all individuals sampled; following the definition of Turnbaugh et al., 2007), than for variable microbiota (i.e., not present in all samples). Our findings have implications for improving ongoing restoration programmes by considering microbial associations that may enhance restoration success by improving the resilience and functioning of restored ecosystems.

## 2 MATERIAL AND METHODS

### 2.1 Sampling

**FIGURE 1:**
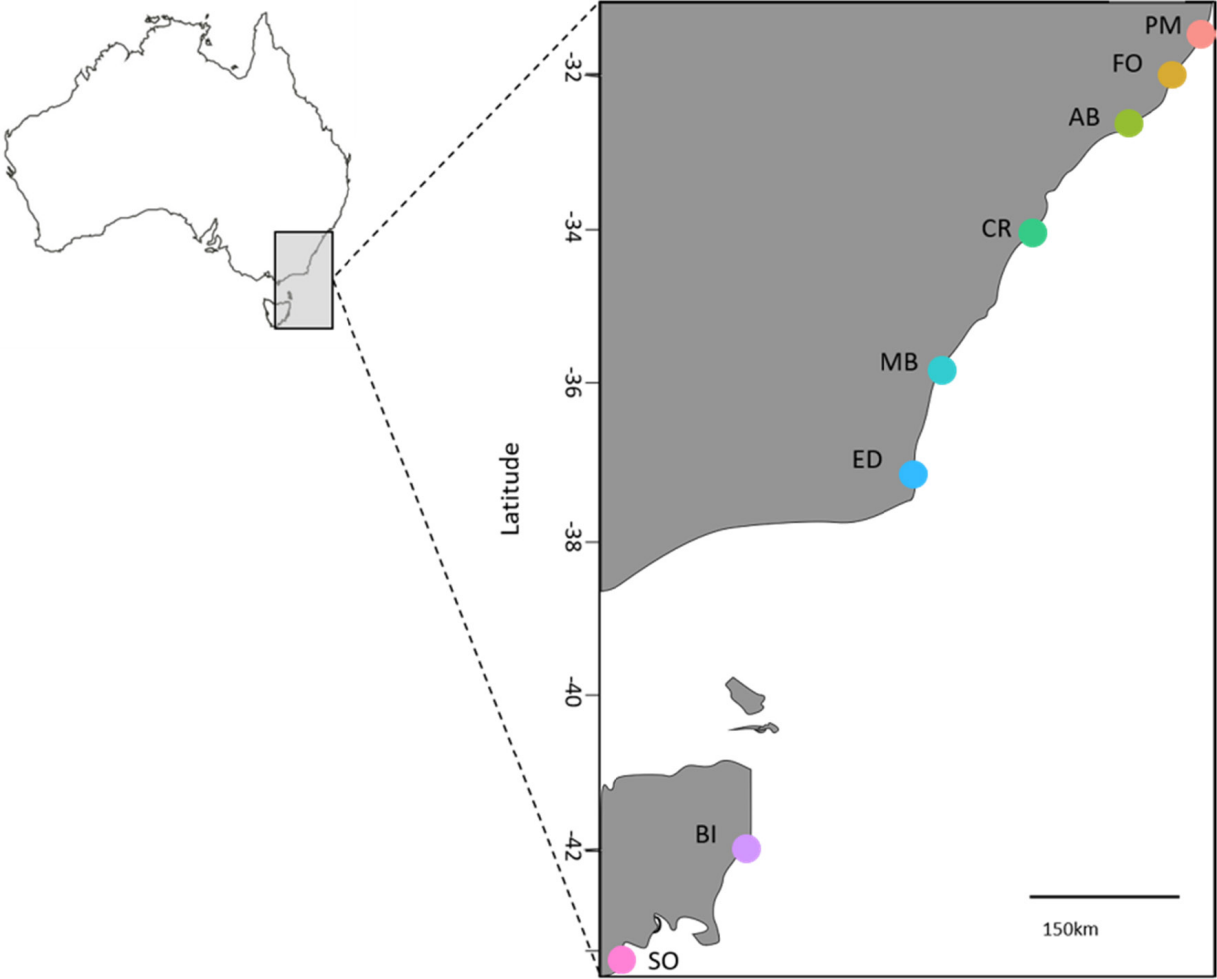
Map of sites where the seaweed *Phyllospora comosa* was sampled for a study on host genetics, phenotype and surface‐associated microbial communities: PM, Port Macquarie (*n* = 20); FO, Forster (*n* = 20); AB, Anna Bay(*n* = 19), CR, Cronulla (*n* = 19); MB, Malua Bay (*n* = 19); ED, Eden (*n* = 20); BI, Bicheno (*n* = 20); SO, Southport (*n* = 19)

We sampled *Phyllospora* individuals and their associated microbial communities at eight rocky reef sites spanning *Phyllospora's* entire latitudinal distribution (~1300 km of linear coastline; Figure 1) along southeastern Australia. Sites were visited in random order over the Austral summer (January) of 2018, to avoid temporal correlations with geographical distribution. At each site, 20 *Phyllospora* adults >1 m apart were haphazardly collected from an area of 500 m^2^ at 1–5 m depth. Sections of 20 cm were cut from the middle of the thallus of each *Phyllospora* individual and placed into a press seal bag whilst underwater. On the surface, samples were kept in their bags on ice for up to 30 min, rinsed with 0.22‐μm filtered seawater to remove any unattached epibionts and a sterilized cotton swab was used to sample the surface (Marzinelli et al., 2015). Swabs were swiped firmly over 25 cm^2^ of healthy frond tissue for 30 s before being stored immediately in liquid nitrogen, transported to UNSW Sydney and kept at −80°C until DNA extractions were performed.

**FIGURE 2:**
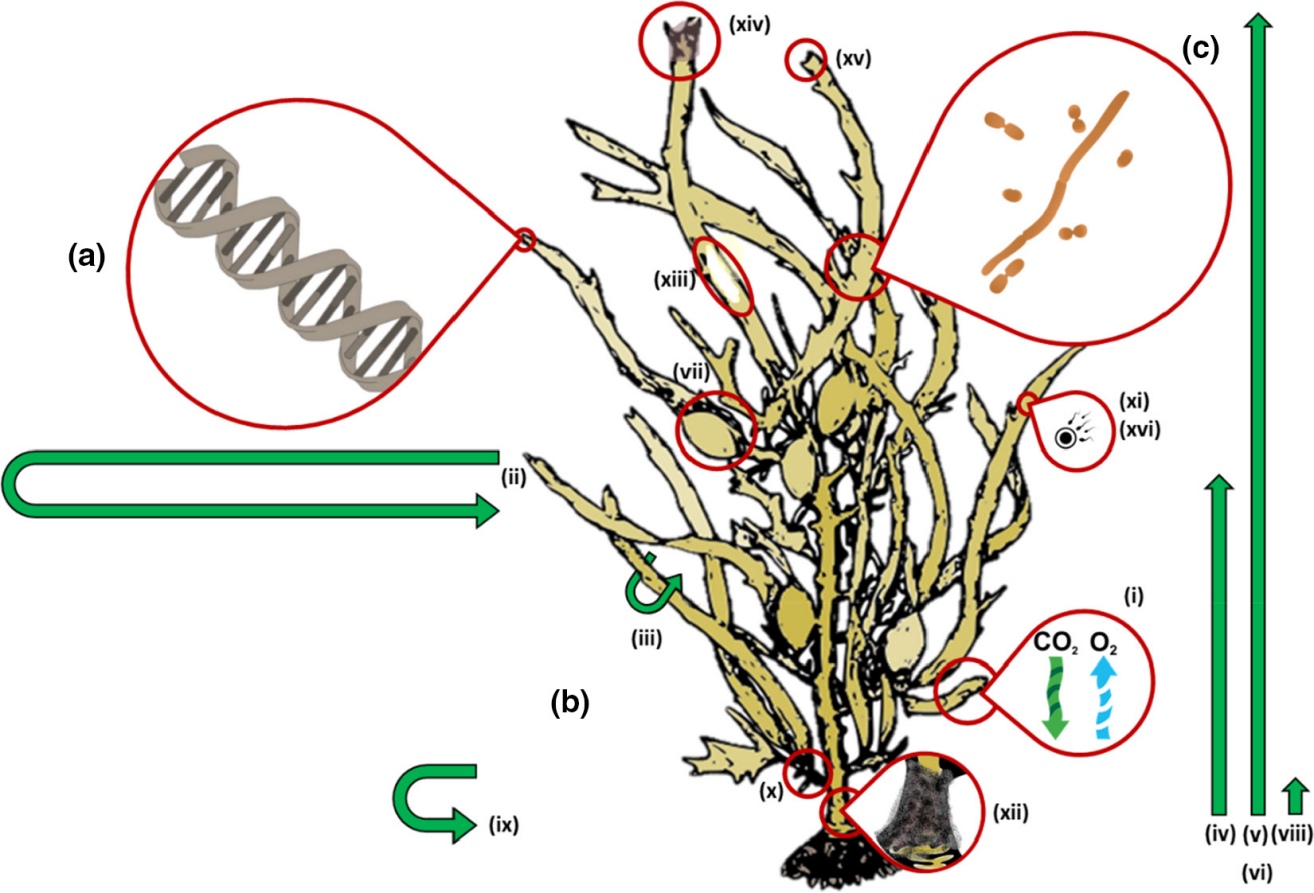
Traits measured or characterized for each of 156 *Phyllospora comosa* individuals. (a) Host genetics; (b) phenotypic traits, including (i) maximum photosynthetic quantum yield, (ii) thallus circumference, (iii) frond width, (iv) primary length, (v) total length, (vi) wet weight, (vii) number of vesicles, (viii) stipe base length, (ix) stipe base width, (x) number of branches, (xi) density of reproductive conceptacles, (xii) presence of stipe rot disease, (xiii) percentage of thallus bleached, (xiv) percentage of thallus fouled by epibionts, (xv) scaled presence of grazing and (xvi) sex; and (c) microbial communities

We characterized each individual seaweed's morphology by quantifying wet weight (biomass), length of the thallus (the length from the top of the stipe to the longest apical tip), total length (including secondary fronds), thallus circumference, frond width, stipe width and stipe length (Figure 2). *Phyllospora* is a dioecious species; we recorded sex (male/female) and the mean number of reproductive conceptacles found within a 25‐mm^2^ quadrat placed over three randomly selected fronds to estimate reproductive capacity. We assessed photosynthetic capacity as a proxy for host functioning by quantifying maximum quantum yield of one dark‐adapted leaf per individual using a Pulse Amplitude Modulated (PAM) fluorometer (WALZ), and seaweed condition by visually estimating levels of herbivory (presence of bite‐marks and missing fronds on a scale of zero to four, four being very highly grazed), bleaching (percentage of the thallus with a distinctive lighter coloration characteristic of bleaching disease), biofouling (percentage of thallus covered with attached Bryozoa or filamentous epiphytes) and the presence of putative disease (stipe rot, diagnosed by the presence of black, spongey stipe tissue above the holdfast; see Campbell, Marzinelli, et al., 2014; Ferrari et al., 2021 for further sampling details).

Ten unfouled apical tips were removed from each individual for genotyping. Samples were rinsed in fresh water and dried to remove external salt, epiphytes and water (see Coleman & Brawley, 2005). Samples were snap‐frozen in liquid nitrogen and stored at −80°C.

### 2.2 Processing and bioinformatics

#### 2.2.1 Microbial community samples

DNA was extracted in random order from each swab sample using the Powersoil DNA Isolation Kit (Mo Bio Laboratories #12888‐100) following the manufacturer's guidelines. DNA extracts were stored in a −20°C freezer until amplification with polymerase chain reaction (PCR). We used the primers 341F (5’‐CCTACGGGNGGCWGCAG‐3’) and 805R (5’‐GACTACHVGGGTATCTAATCC‐3’), which target V3–V4 regions of the 16S rRNA gene (Klindworth et al., 2013). Agarose gel electrophoresis, Nanodrop 1000 and the Qubit 2.0 Fluorometer (Thermo Fisher Scientific) were used to check the quality and quantity of the amplicons before being sent to the Ramaciotti Centre for Genomics (UNSW) for sequencing via the Illumina MiSeq 2000 Platform.

Gene sequence reads were quality filtered using trimmomatic (Bolger et al., 2014) with a sliding window trim of 4:15 bp and removal of sequences with <36 bp. Paired‐end reads were merged with a minimum length of 400 bp and maximum of 500 bp using usearch (Edgar, 2010). unoise was then used to remove chimeras and produce amplicon sequence variants (ASVs), that is operational taxonomic units at a unique sequence level (0% distance) (Edgar, 2016). usearch was used to map the original reads back to ASVs, generating a table of 3170 ASVs. ASV sequences were searched with blastn against the SILVA SSU Ref NR99 database for taxonomic classification. ASVs assigned to chloroplasts and rare ASVs (<0.1% total abundance) were removed. We standardized individual counts by their respective sample sequencing depth (total number of ASV counts) to obtain relative abundances and this data set was used for compositional and diversity statistical analyses. For analyses of individual ASVs, we used raw counts normalized using deseq2 (McMurdie & Holmes, 2014).

#### 2.2.2 Host genetics

Frozen frond tips (~25 mg) were ground to a powder in a Qiagen Tissuelyser 2000 using stainless steel beads without thawing. DNA was extracted and SNPs were sequenced as in Wood et al. (2020).

Subsequent bioinformatics and data analyses were conducted using the statistical platform R (version 3.6; R Core Team, 2019). We quality filtered the data, excluding SNPs and samples with a call rate below 90% of the total, or with a minor allele frequency (MAF) below 0.05. We also filtered for linkage disequilibrium using *SNPrelate* (Zheng et al., 2012) with a threshold value of 0.7, which removed two loci from the data set. Exact tests for deviations from Hardy–Weinberg equilibrium (HWE) were calculated across all samples and loci in the data set using *hierfstat* (Goudet, 2005) and corrected for multiple testing using the Benjamini–Hochberg false discovery rate (FDR) procedure (Benjamini and Hochberg, 1995). Out of 115 loci, 45% deviated from HWE, but only at one or two sites, so these loci were retained in the data set. One locus was identified as deviating from HWE at 10 (77%) sites and exhibited high heterozygote deficiencies (*F*_IS_ =0.918). We removed this locus as this is probably due to null alleles or other genotyping errors (Hosking et al., 2004), which left a total of 114 loci out of the original 354.

### 2.3 Statistical analyses

All analyses were conducted using the R statistical platform (version 3.6; R Core Team, 2019). We first calculated descriptive statistics including (i) how much genetic and phenotypic variation there was amongst sites and individuals; (ii) correlations between geography, phenotype and genetics; (iii) how many ASVs were identified across all individuals in total; and (iv) how many ASVs were shared by all individuals (putative “core” taxa) vs. those that were not shared by all individuals (“variable” taxa).

Genetic and phenotypic data were first visualized using principal component analysis (PCA) ordinations. Distance matrices for genetic allele frequencies and normalized phenotypic data (including all 16 traits) were calculated based on Euclidean distances between samples using the *vegdist* function. We then assessed differences in genetic variation among the three broad clusters identified in these visualizations (geographical “regions” corresponding to rear, central and leading‐edge populations) and sites within these clusters using analysis of molecular variance (AMOVA) in poppr. Phenotypic variation among sites was also tested using permutational multivariate analysis of variance (PERMANOVA; Anderson, 2001) with the *adonis* function in *vegan* (999 permutations; Oksanen et al., 2018). Variance components were used to determine the amount of variation due to Site. Regions/clusters explained a significant but small percentage of the genetic variation in comparison to sites and samples within sites (see Results), and thus we conducted all further analyses comparing individuals among (i.e., over the entire latitudinal scale) or within sites (site‐level scale). Post‐hoc pairwise tests between sites were performed with 999 permutation and with an FDR (Benjamini & Hochberg, 1995) to correct for multiple comparisons. We used permutational multivariate dispersion analyses (*betadisper*) to test for homogeneity of multivariate dispersion among sites (Anderson, 2006), followed where necessary by post‐hoc tests (corrected using the Tukey method) using the *emmeans* package (Lenth, 2018).

To examine relationships between host genetics/phenotype and geography, the correlation between their respective Euclidean distance matrices was tested via Mantel tests in *vegan*. We also tested for differences in individual phenotypic traits between sites using a series of linear and (where data conformed to a binomial distribution) generalized linear models. Correlations between host phenotypic traits and latitude were tested using the Spearman correlation coefficient and FDR adjustment for multiple comparisons.

We examined the relationship between microbial communities and geography by comparing differences in microbial communities between sites with PERMANOVA. To better understand how host and geographical factors influence different components of the microbiota, we conducted a subset of the statistical analyses on the overall, core and variable taxa data sets separately. Similarity matrices were calculated based on Bray–Curtis distances on square‐root‐transformed relative abundance data (“community structure”). Analyses used 999 permutations and data were visualized using nonmetric multidimensional scaling (nMDS) ordinations. Post‐hoc pairwise tests were performed with 999 permutations and an FDR was applied to correct for multiple comparisons. We used permutational multivariate dispersion analysis (*betadisper*) to test for homogeneity of multivariate dispersion within groups (Anderson, 2006), followed where necessary by post‐hoc tests with an FDR applied to correct for multiple comparisons. We further tested for correlations between microbial community dissimilarity (based on the centroid of the Bray–Curtis dissimilarity matrix) and geographical distance between sites with Mantel tests in *vegan* and again applied an FDR correction to assess significance.

To examine the relationship between microbial community structure and host genetics at a site level, we calculated host genetic diversity (expected heterozygosity, *H*_E_) per site with *DartR* (Gruber et al., 2018) and estimated microbial community alpha diversity using species richness and Simpson's diversity indices calculated with *vegan*. We then tested for relationships between population‐level genetic diversity (*H*_E_) and community alpha‐diversity at each site via linear regression.

To determine if there was a relationship between phenotypic or genetic distance and microbial community dissimilarity (beta diversity) between individual samples, we calculated genetic and phenotypic (Euclidean) distances between hosts and Bray–Curtis dissimilarity matrices on square‐root‐transformed relative abundances for all microbiota samples with the *vegdist* function. We then tested for correlations between (i) genetic diversity and microbial community beta‐diversity and (ii) phenotypic diversity and microbial community beta‐diversity across all sites using Mantel tests in *vegan*, using an FDR correction to assess significance. As there was a significant correlation between geographical distance and host genetic distance between sites (see Results), genetic data were analysed using partial Mantel tests, which first accounted for the effects of geography (Euclidean distance between samples, calculated using site‐level coordinates). We also ran an additional series of Mantel tests that tested for correlations between the factors above using only comparisons between hosts within each site. Overall, these analyses allowed us to assess the relationship between host genetic/phenotypic differentiation and microbial community dissimilarity at both continental (1300 km linear) and local (500 m^2^) scales.

To identify specific host traits that may be associated with microbial community structure, we performed distance‐based linear models and redundancy analyses (dbRDA; McArdle & Anderson, 2001) on the Bray–Curtis dissimilarity matrix of square‐root‐transformed ASV relative abundances in *vegan*, including the factor Site first in the model to account for geographical effects. Variables that were strongly correlated (*r* ^2^ > .7) were removed, leaving one in the set to represent those removed. To determine the variance explained by geographical, genetic and phenotypic aspects separately, we first fitted the dbRDA model on each component. We then identified which of these aspects explained microbial communities overall (using the overall data set), identifying the most parsimonious model including all possible variables using model selection with a stepwise procedure (direction = both) based on *p*‐values. Missing phenotype data (0.9% of the data) were imputed with the average of each metric at each site. Allele frequencies were imputed by using the most common allele frequency observed within each genetic cluster using *sNMF* (Frichot et al., 2014) in the *LEA* (Frichot & François, 2015) package.

To determine which ASVs were associated with the host traits selected by the dbRDA model above, we fitted multivariate generalized linear models in deseq2 assuming a negative‐binomial distribution for each ASV. To confine the number of models to an easily interpretable number and because the influence of genetics, phenotype and geography over core and variable taxa was similar in all previous analyses (see Results), we fitted these models on the overall microbial data set only. There were seven variables identified as important using model selection; each variable was fitted as a single predictor in separate models, which also included the factor Site first in the model to account for geographical effects. Likelihood ratio tests were used to test for the significance of each variable, using adjusted *p*‐values to account for multiple testing. Prior to analyses, raw ASV count data were normalized with size factor dispersions calculated for each treatment combination to account for differences in sequencing depth (Love et al., 2014). Dozens of taxa turned out to significantly differ among each of the variables, so we only focused on the five most abundant ASVs that were significantly affected by each variable using alpha =.01. Wald pairwise tests were used to compare relative abundances of taxa among categorical treatments with adjusted *p*‐values to account for multiple testing.

## 3 RESULTS

### 3.1 Host traits

**FIGURE 3:**
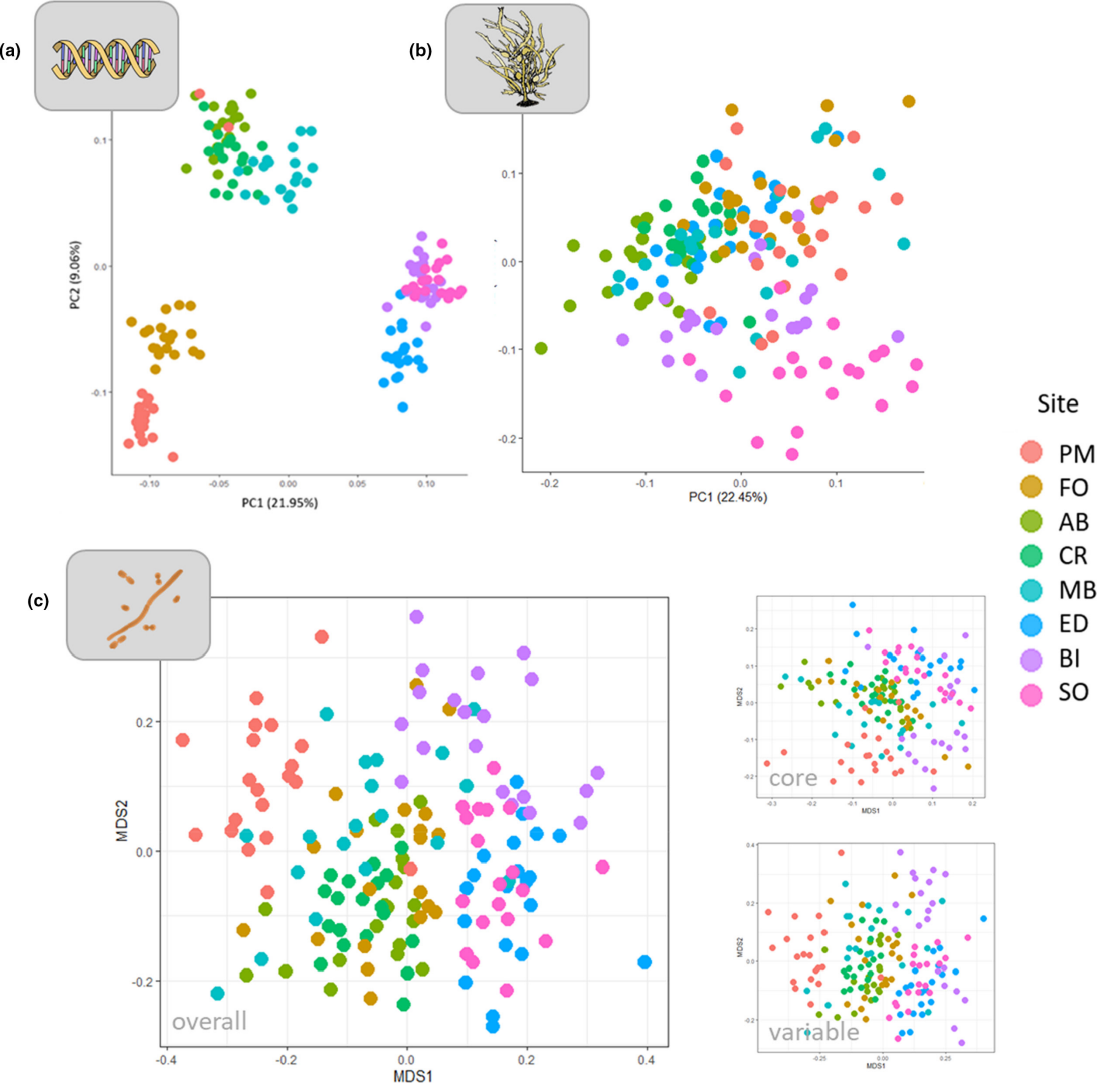
(a) Principal component analysis (PCA) of the seaweed *Phyllospora comosa's* genetic structure, based on allele frequencies at 114 SNP loci; (b) PCA of phenotype, based on 11 traits describing morphology and condition; (c) nMDS analysis of *Phyllospora*‐associated microbial communities (overall community and core and variable taxa shown separately), based on Bray–Curtis dissimilarity matrices for square‐root‐transformed relative abundances, Sites, north to south: PM, Port Macquarie (*n* = 20); FO, Forster (*n* = 20); AB, Anna Bay(*n* = 19), CR, Cronulla (*n* = 19); MB, Malua Bay (*n* = 19); ED, Eden (*n* = 20); BI, Bicheno (*n* = 20); SO, Southport (*n* = 19)

A significant amount of genetic variation was explained by geographical region (AMOVA, 14.32%, *p* = .01, Table SI.1) and sites (AMOVA, 22.08%, *p* = .01), while the majority of genetic variation occurred among individuals (AMOVA, 63.46.8%, *p* = .03). Overall, genetic diversity was low at all sites (*H*_E_: 0.179–0.336); however, host genetics varied significantly among sites, with 50% of the genetic variation explained by site‐to‐site variation (*F*_7,148_ = 21.02, *p* = .01; Figure 3a and Table S2) and three clusters visible on the PCA. There were also differences in the dispersion of genetic data between sites (permdist *F*_7,148_ = 16.95, *p* = .01, Figure S1 and Table S3).

Host phenotype varied significantly between sites, with 31% of the total phenotypic variation explained by site‐to‐site variation (*F*_7,148_ = 9.51, *p* = .01; Figure 3b). Only two pairs of sites were not phenotypically different from each other when considering overall phenotype (Eden and Bateman's Bay, *p* = .24, Forster and Port Macquarie, *p* = .12, Table S4), but there were differences in the dispersion of phenotypic data between sites (*F*_7,148_ = 3.96, *p* = .01, Figure S2), with dispersion in one site (Cronulla) significantly lower than at the other sites (Table S5). Individually, all phenotypic traits except total number of branches varied significantly between sites (Figure S3, Table S6). Several morphological traits were significantly correlated with each other and with latitude (Table S7). Broadly, the total length of individuals was much greater at the Tasmanian (cooler range‐edge) populations. Maximum photosynthetic quantum yield was highest at Bicheno, Southport and Anna Bay. Evidence of bleaching and thallus fouling was found across all sites and was relatively high on average (~20%) at Anna Bay. Evidence of herbivory/grazing was highest at Bicheno. Stipe rot disease was also present at all sites except for Port Macquarie and was also highest at Anna Bay.

### 3.2 Microbial communities

There were 3,170 unique ASVs found across all 160 samples. These were reduced to 2061 ASVs following filtering and removal of samples where host DNA did not amplify (i.e., to correspond to the 156 genotyped samples). The “core” microbial community was represented by 23 ASVs (1.1% of all ASVs), which were found in every sample and represented an average of 45.2% (*SE*: 1.3) of the relative abundance within the community. Most of these (60%) belonged to the phylum Proteobacteria; the phyla Verrucomicrobia, Planctomycetes and Cyanobacteria were also present (Table S8). These taxa represented seven genera: *Arenicella* and *Granulosicoccus* (class Gammaproteobacteria), *Blastopirellula* (class Planctomycetacia), *Hellea*, *Litorimonas* and three other uncultured strains of the class Alphaproteobacteria, *Rubritalea* (class Verrumicrobiae) and an Oxyphotobacteria (Table S7).

### 3.3 Host–microbiota relationships

There was a significant effect of site on microbial community structure and composition for the overall, variable and core microbiome (overall: *F*_7,148_ = 11.37, *p* = .01 and *F*_7,148_ = 7.64, *p* = .001; variable: *F*_7,148_ = 11.162, *p* = .001 and *F*_7,148_ = 7.30, *p* = .001; core: *F*_7,148_ = 11.063, *p* = .001 and *F*_7,148_ = 8.87, *p* = .001; Figure 3c,d). For the overall and variable microbiome, there were differences between all sites (Table S9a,b), while the core microbiome at Anna Bay and Cronulla were not significantly different (Table S7c). There were also differences in the dispersion of microbial community structure and composition between some sites (overall: *F*_7,148_ = 3.83, *p* = .001, *F*_7,148_ = 4.03, *p* = .001; variable: *F*_7,148_ = 6.35, *p* = .001, *F*_7,148_ = 4.035, *p* = .001; core: *F*_7,148_ = 3.01, *p* = .006, *F*_7,148_ = 3.25, *p* = .002; Figure S4a–c), with dispersion in one site (Cronulla) again being generally lower than at other sites (Table S10).

**FIGURE 4:**
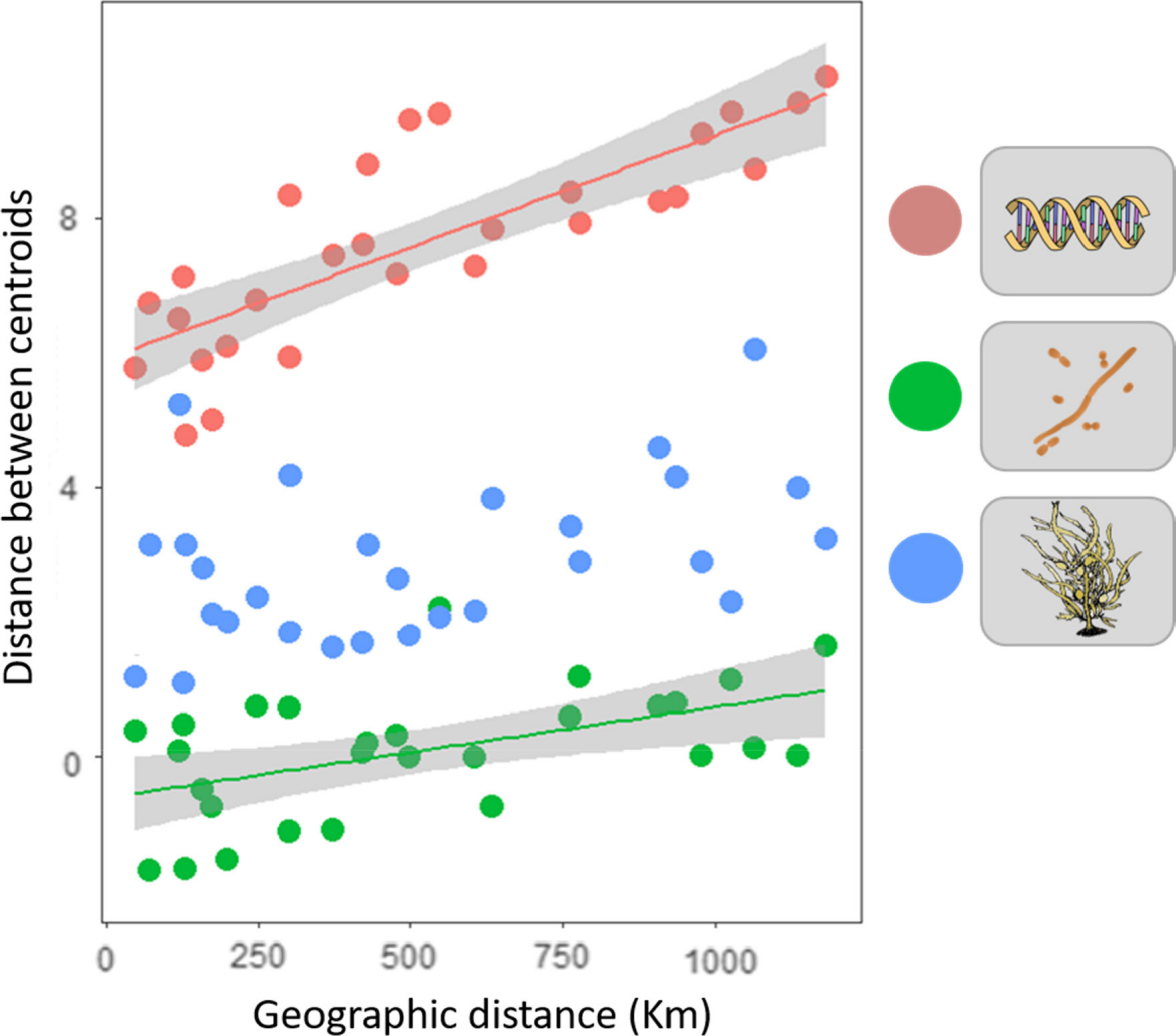
Relationship between geographical distance (km) and pairwise distance/dissimilarity between centroids of the eight *Phyllospora comosa* populations for genetics (red symbols; Euclidean distances), overall surface‐associated microbial communities (green symbols; Bray–Curtis dissimilarity of square‐root‐transformed relative abundances) and phenotype (blue symbols; Euclidean distances). Data with significant associations detected using Mantel tests were fitted with linear regression. 95% Confidence intervals are shaded in grey

Host genetic distance among sites and overall microbial community dissimilarity were significantly correlated with geographical distance (*p* = .01, *r* = .78; *p* = .05, *r* = .51, respectively; Figure 4), while phenotypic distance was not (*p* = .06, *r* = .43; Figure 4).

There was no significant relationship between the genetic diversity of host populations (*H*_E_) and rarefied overall microbial ASV species richness or average Simpson diversity per sample at each site (*F*_1,6_ =.57, *p* = .48, Figure S5a and *F*_1,6_ = 1.00, *p* = .36, Figure S5b, respectively). Results were similar for both the core (*F*_1,6_ =.659, *p* = .659; Simpson diversity results only) and variable (*F*_1,6_ = 1.098, *p* = .335 and *F*_1,6_ = .604, *p* = .604, respectively for species richness and Simpson diversity) components of the microbial community.

**FIGURE 5:**
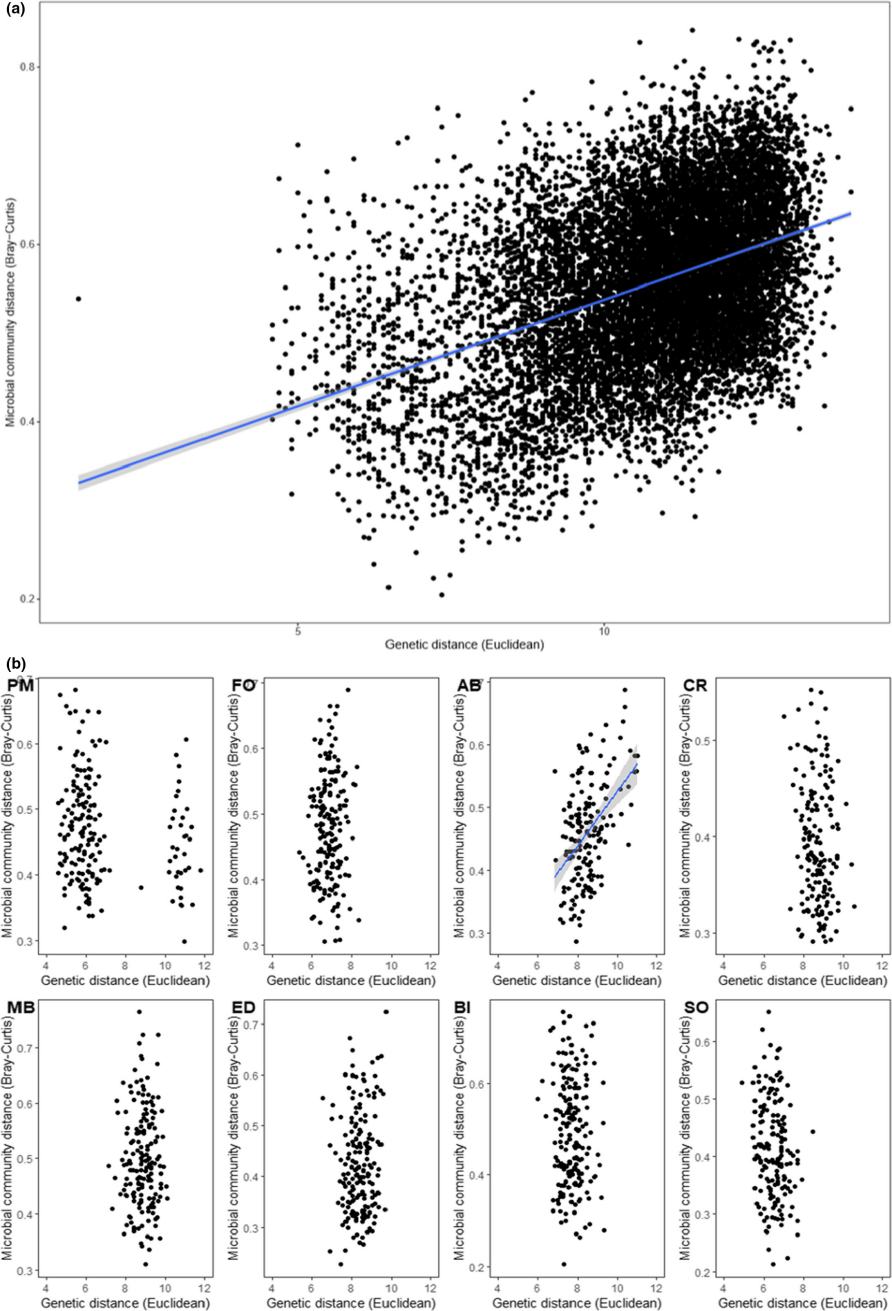
Relationship between microbial community dissimilarity (Bray–Curtis on square‐root‐transformed data) based on all ASVs and genetic distance (Euclidean) between all *Phyllospora comosa* individuals (a) across and within all sites, and (b) within each site. Sites are ordered north to south: PM, Port Macquarie (*n* = 20); FO, Forster (*n* = 20); AB, Anna Bay (*n* = 19), CR, Cronulla (*n* = 19); MB, Malua Bay (*n* = 19); ED, Eden (*n* = 20); BI, Bicheno (*n* = 20); SO, Southport (*n* = 19). Data with significant associations detected using partial Mantel tests were fitted with linear regression. 95% Confidence intervals are shaded in grey

**FIGURE 6:**
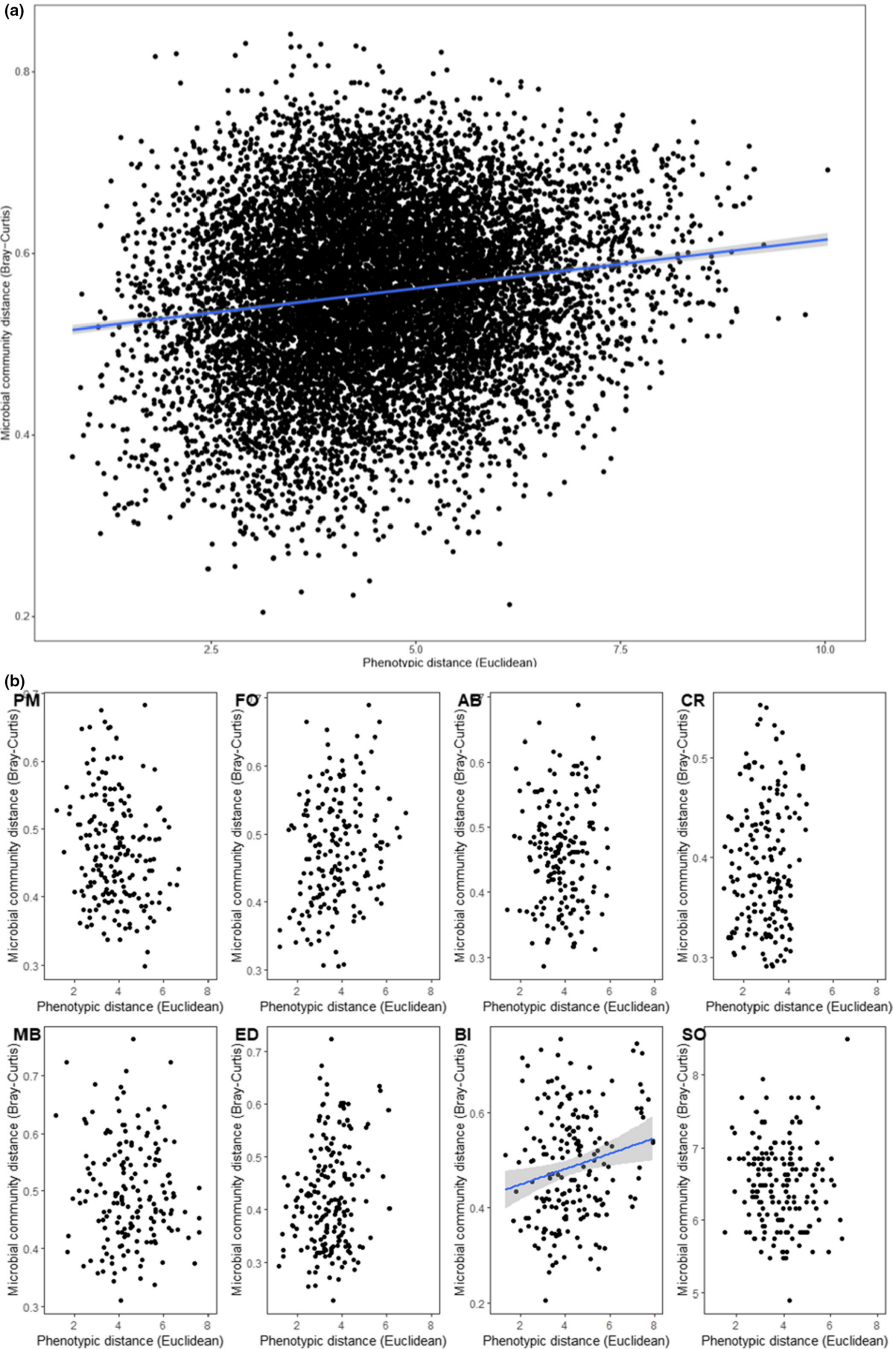
Relationship between microbial community dissimilarity (Bray–Curtis on square‐root‐transfored relative abundances) based on all ASVs and phenotypic distance (Euclidean) between all *Phyllospora comosa* individuals (a) across and within all sites, and (b) within each site. Sites are ordered north to south: PM, Port Macquarie (*n* = 20); FO, Forster (*n* = 20); AB, Anna Bay (*n* = 19), CR, Cronulla (*n* = 19); MB, Malua Bay (*n* = 19); ED, Eden (*n* = 20); BI, Bicheno (*n* = 20); SO, Southport (*n* = 19). Data with significant associations detected using Mantel tests were fitted with linear regression. 95% Confidence intervals are shaded in grey

Pairwise host genetic distance and overall microbial community dissimilarity were positively correlated overall (partial Mantel, *p* = .001, *r* = .2411, Figure 5a). Within sites, however, only one location (Anna Bay) exhibited a significant correlation between genetic distance and overall microbial community dissimilarity (Figure 5b; Table S11). Phenotypic distance and overall microbial community dissimilarity were also positively correlated overall (Mantel, *p* = .01, *r* = .15, Figure 6a). Within sites, however, only one location (Bicheno) maintained a significant correlation between phenotypic distance and overall microbial community dissimilarity (Figure 6b; Table S11). Results for variable taxa were similar to overall community data (partial Mantel, *p* = .001, *r* = .2235; Table S11), whilst when the core microbial taxa were considered only, slightly less variance was explained by genetics (partial Mantel, *p* = .001, *r* = .1425). Within sites, however, Cronulla also exhibited a significant correlation between genetic distance and core microbial community dissimilarity, and Eden, but not Bicheno, exhibited a correlation between phenotypic distance and core community dissimilarity (Table S11).

**FIGURE 7:**
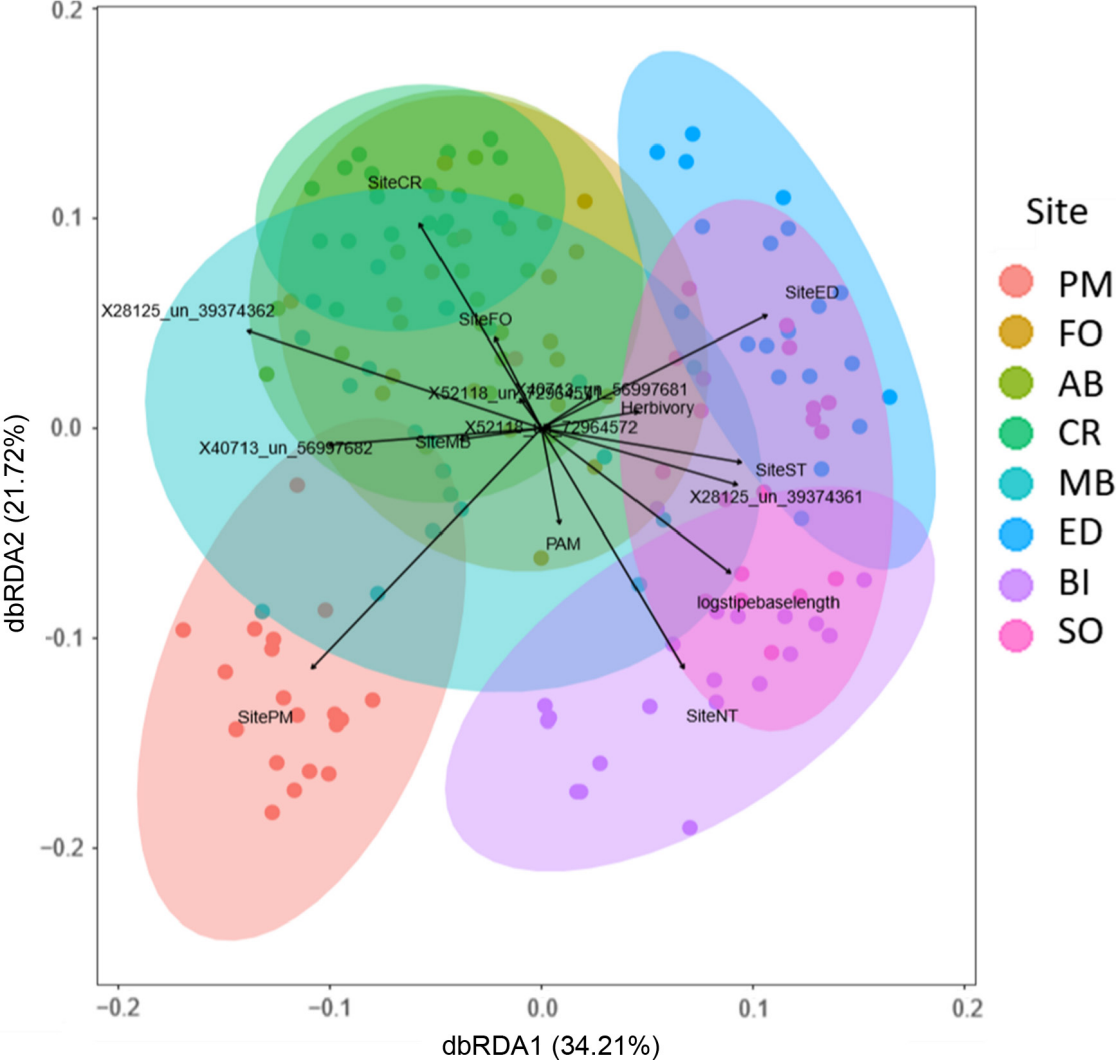
Associations between geography, genetics and phenotype of the *Phyllospora comosa* host with 156 microbial community samples isolated from the surface of host fronds, as inferred by distance‐based redundancy analysis (dbRDA). Vectors indicate the direction and strength (length) of each significant variable in explaining variation between microbial community samples. Vector labels starting with “site” indicate a specific site association; PAM = maximum quantum yield; logstipebaselength = stipe base length (data log‐transformed), Herbivory = amount of grazing on thallus; all vectors starting with X indicate SNP loci. Numbers at the end of SNP names indicate allele frequencies of the major allele at that locus. PM, Port Macquarie (*n* = 20); FO, Forster (*n* = 20); AB, Anna Bay (*n* = 19), CR, Cronulla (*n* = 19); MB, Malua Bay (*n* = 19); ED, Eden (*n* = 20); BI, Bicheno (*n* = 20); SO, Southport (*n* = 19)

Individual dbRDA models revealed that Site explained 32.57% of overall, 31.9% of variable and 33.3% of core microbial community variation. Site +all SNPs explained 53.63%, 55.3% and 64.3% while Site +all phenotypic traits explained 34.9%, 34.6% and 36.2% of overall, variable and core variation, respectively (Table 1). After model selection, the final dbRDA model of all ASVs including the covariates site, maximum quantum yield, herbivory, stipe base length and three SNP loci (28125_un_3937436, 40713_un_5699768 and 52118_un_7296457) explained a significant amount (35%) of the variation in overall microbial community structure. Of these covariates, site‐specific differences and allele frequencies at locus 28125_un_3937436 had the greatest influence on microbial communities (Figure 7).

**TABLE 1:** ANOVA output table for (i) initial and (ii) final model‐selected dbRDA models, showing geographical, host phenotypic and host genetic associations with *Phyllospora comosa's* associated microbial community structure. The final model was selected via model selection based on *p*‐values, using a stepwise selection procedure

Model called	*df*	SS	*F*	*p*	Adjusted *R* ^2^ overall	Adjusted *R* ^2^ variable	Adjusted *R* ^2^ core
(i)							
Site					.326	.319	.333
Phenotype					.115	.111	.114
SNPs					.536	.553	.643
Site + phenotype					.349	.343	.362
Site + SNPs					.536	.553	.643

Model called	*df*	SS	*F*	*p*	Adjusted *R* ^2^ overall
(ii)					
Overall ASVs ~Site + PAM + 28125_un_3937436 + 40713_un_5699768 + Herbivory + Stipe base length + 52118_un_7296457	16	10.36	6.302	.001	.350
	139	14.28			

Based on the overall microbiome data set, geography (differences between sites) influenced a large number of ASVs (1199), the most abundant of which were assigned to the classes Planctomycetacia, Oxyphotobacteria and Alphaproteobacteria (Table S12 and Figure S6a). A comparatively lower number of ASVs were associated with individual SNPs than with geography (13–50 ASVs associated with each SNP locus, vs. >1000 ASVs related to differences between sites; Table S12). The most abundant ASVs associated with specific loci were assigned to the classes Planctomycetacia, Bacteroidia, Proteobacteria, Oxyphotobacteria, Verrucomicrobia, Gammaproteobacteria and Alphaproteobacteria and the family Caldilineaceae (Figure S6b).

Overall, phenotypic traits were associated with the least number of ASVs relative to other model covariates (13–31). Of those ASVs from the overall data set that were associated with phenotypic traits, the most abundant were assigned to the classes Bacteroidia, Proteobacteria, Verrucomicrobia, Gammaproteobacteria and Alphaproteobacteria (Table S12 and Figure S6c). Pairwise test results are presented in Supporting Information 2.

## 4 DISCUSSION

The importance of microbial communities to the development, health and survival of eukaryotic hosts is a key emerging theme in contemporary ecology. Understanding the mechanisms underpinning these interactions is critical, particularly in the context of environmental change, species declines, and the development of active restoration and future‐proofing strategies (Breed et al., 2019; van Oppen & Blackall, 2019; Wood et al., 2019). This study investigated the relationship between geography, host genetics and phenotypic traits and variation in the microbiome of a forest‐forming seaweed, *Phyllospora comosa*. Using these multifaceted data, we were able to explain up to two‐thirds of the microbial community variation even at a high taxonomic resolution (ASVs). In most cases, *Phyllospora's* microbiota was most strongly associated with local environmental conditions (i.e., was site‐specific). Nevertheless, similar host genotypes and phenotypes generally had more similar microbial communities, with the effect of these traits on community dissimilarity being most apparent at regional, rather than site‐specific, scales.

There were strong correlations between *Phyllospora* genetic structure and geography, which was unsurprising given that many macroalgae including *Phyllospora* are limited in their dispersal and exhibit strong genetic isolation‐by‐distance along the coast (Durrant et al., 2015; Wood et al., 2020; 2021). Nevertheless, our modelling approach allowed us to examine the respective influences of these two factors on overall microbial communities. For example, partial Mantel correlations, which accounted for the influence of Site, indicated that host genetic distances explained 24% and 14% of the overall and core microbial community dissimilarity, respectively. Meanwhile, dbRDA showed that host phenotype explained slightly more variation than Site alone (2.3% and 3.1% for the overall and core microbial community, respectively). Using SNPs combined with ecological data enabled us to identify key loci and phenotypic traits that could be used to predict the relative abundance of specific ASVs found on *Phyllospora's* surface, including taxa with known associations to seaweed disease and cell degradation. Our results indicate that *Phyllospora's* surface‐associated microbial communities are jointly shaped by local environmental conditions and host‐specific differences. The strength of each of these factors is, however, context‐specific, and may be dependent on trade‐offs between each of these (e.g., the direction and strength of the influence of ecological interactions such as herbivory vs. genetics) and additional ecological or environmental influences (e.g., Marzinelli et al., 2015).

### 4.1 Composition of *Phyllospora's* microbiome

Although *Phyllospora's* microbial communities varied widely, we found that almost half of the bacteria present (~45% of the total relative abundance) consisted of just 23 ASVs that were found on every individual. This level of similarity amongst individuals is at least one order of magnitude greater than has been found for other holobionts, such as corals (Hernandez‐Agreda et al., 2018), and humans (Rothschild et al., 2018), but similarly high levels of microbiome homogeneity have been found for other seaweeds (e.g., Roth‐Schulze et al., 2018) and also for sponges (e.g., Easson et al., 2020; Marino et al., 2017; but see Griffiths et al., 2019).

While the particular functional roles of the taxa found here remain to be explored via experimental means, many of the core genera have been demonstrated to be probably core taxa across seaweeds broadly. For example, *Blastopirellula*, *Hellea* and *Litorimonas* have been widely found to be some of the most abundant taxa present on brown seaweeds (Brunet et al., 2021; Ihua et al., 2020; James et al., 2020; Quigley, 2018; Vollmers et al., 2017; Weigel & Pfister, 2019). *Rubritalea* is also emerging as a “core” genus present in seaweed across all life stages from early development (Han et al., 2021). Several of these taxa (*Hellea*, *Litorimonas*, *Granulosicoccus*) have also been directly associated with healthy, rather than degraded, tissue and thus probably play a key role in healthy macroalgal microbiomes, with changes in relative abundance potentially serving as indicators of host stress and degradation (Brunet et al., 2021; James et al., 2020). Considering the stronger association with core taxa and host genetics when compared to the overall microbiome or variable taxa, it is possible that these taxa are involved in co‐adapted relationships with their seaweed hosts. Further research focusing on this and decoupling the influences on variable taxa could be critical for understanding the biology of *Phyllospora* and other foundational seaweed species.

### 4.2 Influence of geography on microbiome

We found a strong association between geography and the structure and composition of *Phyllospora's* microbial communities. Differences in the overall microbiome, as well as core and variable components, were linked to both large‐scale patterns that could be attributed to geographical distance between sites, and also site‐to‐site differences. Teasing apart the specific environmental drivers of these patterns was beyond the scope of this study, but previous work has highlighted that temperature, light (Brown et al., 2012; Ghiglione et al., 2012; Gilbert et al., 2012; Rusch et al., 2007), salinity (Weigel & Pfister, 2019), wave motion and phosphate concentrations (Marzinelli et al., 2015), and habitat structure (Marzinelli et al., 2018) can be influential over small (metre) and medium (kilometre) spatial scales. It is important to note that some driving factors may be more important than others; many of the differences between sites were attributed to changes in abundance of ASVs that have been previously linked to seaweed defence (Hyphomonadaceae), heat stress (Blastopirellula) and cell‐wall degradation (Cyanobacteria; Hollants et al., 2013). This suggests that local‐scale differences in temperature and grazing may be particularly important drivers of microbial communities in this system.

### 4.3 Genetic associations with microbial communities

There was also a strong correlation (~24% once Site was accounted for) between host genetic distance and microbial community dissimilarity over *Phyllospora's* entire latitudinal distribution, although whether this was causal could not be discerned. At the site level, there was little relationship between host genetic distance and microbial community dissimilarity, which suggests that the associations observed over large latitudinal scales were either simply due to geography or that they break down at local scales. A lack of sufficient host genetic differentiation (whether true or due to limited sampling of differentiated individuals) within sites may be the reason for this, although this is unlikely given patterns observed at the northernmost site (Port Macquarie), which had several very genetically distinct individuals, yet still provided no evidence for linked patterns in microbial community differentiation. Nevertheless, host genetics were significantly correlated with microbial community dissimilarity at some sites (Anna Bay for the overall microbial community, and Cronulla for core taxa only), which indicates that host genetic factors are associated with the microbiota at local scales under certain conditions. Previous work suggests that healthy specimens of seaweeds maintain microbial communities more specific to their environment (McGeoch et al., 2019), but when seaweeds become stressed or bleach this specificity breaks down and communities may become more homogeneous (Marzinelli et al., 2015). As *Phyllospora* hosts at Anna Bay and Cronulla had the highest levels of fouling, bleaching and disease relative to all other sampling locations, genetics may be playing a role in determining resistance or susceptibility to these fouled/disease phenotypes, with effects only becoming observable when”healthy” interactions based on nongenetic (e.g., environmental) factors break down. This could also be because other factors which normally drive community patterns (e.g., local environment, phenotype) may not be as important at these sites, or that host genetic effects on the microbiome are stronger. Indeed, Anna Bay and Cronulla had very low variation in many morphological traits such as length, biomass and photosynthetic activity (Figure S5), so an additional explanation of the patterns observed here could be that when phenotype is less variable, genetic differences become important or are easier to discern.

The idea that genetics may have local context‐specific effects (i.e., genotype/environmental interactions) is further supported by the lack of a detectable link between overall genetic diversity and microbial diversity or species richness at each site. This contrasts with some contemporary ecological theories, which predict that increasing genetic diversity translates to higher phenotypic diversity and available niche space for different species (Evans et al., 2016; Whitham et al., 2006). In this study, a few specific host loci were associated with changes in microbial community structure. Of these, the greatest association was seen at one specific locus (28125_un_3937436) in which being heterozygous or homozygous for the minor allele was associated with higher abundances of several taxa (e.g., the families Saprospiracae and Hyphomonadaceae), including one core taxon (ASV 5; *Hellea*). Taxa from Saprospiracae and Hyphomonadaceae have been linked to warm environmental conditions and increased herbivore associations (Castro, 2019) but lower levels of bleaching disease (Marzinelli et al., 2015) in other species of brown seaweeds. One explanation for the patterns observed could be that this locus and other allele variants across the genome influence community structure via mechanisms that exclude or are beneficial to particular microbial taxa. This is in line with other recent work suggesting that specific environmental or physicochemical characteristics of the host, rather than the overall genetics or host phylogeny, explain microbial community composition (Hacquard et al., 2015; Rothschild et al., 2018). Although we cannot infer causality at this stage, current estimates of overall host genetic associations with microbial diversity that span diverse systems such as sponges (*R* ^2^ = .18–.35, Easson et al., 2020), plants (typical values fall in the range 5%–30%, Bergelson et al., 2021) and mammals (*R* ^2^ ~ .2, Blekhman et al., 2015) are similar, with the contribution of individual alleles expected to consist of small additive variation (as is expected in plant and mammalian systems, Beilsmith et al., 2019). Although we had no specific hypotheses about which ASVs might be affected by differences in alleles, and currently there is no reference genome of *Phyllospora* to map specific locus functions to, our data provide a powerful platform upon which to explore these ideas in the future.

### 4.4 Phenotypic associations with microbial communities

Microbial communities from phenotypically distant hosts were generally more dissimilar over the entire latitudinal distribution, indicating that some of the phenotypic traits measured were associated with microbial community structure. This association was generally quite weak (explained 15% of the variance), but this is to be expected given that our phenotypic measure represented the combination of morphology, disease, photosynthetic efficiency, sex and reproductive capacity—traits which probably had associations of varying direction and magnitude on different microbial taxa. As was the case for genetically dissimilar hosts, however, this association broke down almost completely at local (500 m^2^ within‐site) scales. Phenotypic variation was much lower within sites than between them, potentially presenting insufficient variation to create observable effects. The Bicheno site was the only exception, where phenotypic distance had a significant correlation (22%) with overall microbial community dissimilarity. Phenotypic variation was high at this site, particularly for morphological characteristics and levels of grazing (see Figure SI.5). Phenotype–microbial associations may thus still exist at local scales and may only be observable if (i) other drivers of microbial community structure are weaker, and/or (ii) one or more of the phenotypic associations are strengthened. Future surveys could target these associations by sampling more broadly across phenotypes (we randomly targeted reproductive individuals) or by conducting manipulative experiments with the ASVs associated with herbivory and other phenotypic traits. While we can only speculate about the specific factors contributing to this pattern, it is interesting to note that the Bicheno site had the highest levels of herbivory observed across all locations. Herbivory can be strongly related to microbial community structure in other species of brown seaweeds (Castro, 2019), for example through the deposition of grazer‐specific microbes onto the thallus. In this study, herbivory was one of the key phenotypic variables associated with microbial community composition. Herbivory may thus have stronger associations to the microbiome than other phenotypic/functional traits measured here. Many host or environmental factors were not measured in this study but may also significantly influence microbial communities, such as secretion of specific carbohydrates and proteins (Lachnit et al., 2011; Steinberg and De Nys, 2002), antimicrobial secondary metabolites or differences in photosynthetic yield (which indeed varied across populations). Future studies comparing genetic measures identified here with more localized chemical and isotopic signatures of the hosts may reveal other drivers of host‐associated microbiota (e.g., Bengtsson et al., 2011; Saha et al., 2020; Weigel and Pfister, 2021). Finally, surface‐associated microbial communities can vary between thallus regions (Ihua et al., 2020) and also can change dramatically over the course of development in many organisms (McFall‐Ngai et al., 2013; Yatsunenko et al., 2012). In seaweeds, bacterial communities are known to change according to seaweed age (Weigel & Pfister, 2019). Although we note that the seaweeds sampled within this study were all of a mature age (approximately >1 year, based on the general size and presence of mature vesicles) and thus are likely to be at a mature successional stage (Longford et al., 2019), future work at different stages of succession is warranted.

It is important to note that although we found no significant association between geographical distance and phenotypic distance, seaweed phenotype is generally shaped by localized environmental conditions (Flukes et al., 2015; Fowler‐Walker et al., 2006). Thus, microbial communities that are associated with particular phenotypic traits (e.g., herbivory, stipe length or photosynthetic efficiency) may be confounded by underlying factors that may also shape morphology or the presence of disease phenotypes at local scales (e.g., number of herbivores, temperature, nutrients or wave exposure). This idea is supported by the fact that Site plus phenotype only explained a small amount (~2.3%) of additional variation in microbial community structure than just Site alone, warranting further experimental work to untangle these relationships.

Microbial communities can additionally mediate seaweed traits such as morphology, physiological health and susceptibility to disease or grazing (Campbell et al., 2014). Indeed, many of the most abundant ASVs that were associated with phenotypic traits in this study belonged to genera involved in disease and grazing processes (Hollants et al., 2013; Marzinelli et al., 2015). Mechanistic host phenotype–microbiome associations may also operate in either direction; that is, associated phenotypes may shape microbial community structure, or microbes may be involved in shaping the phenotype of the host, or both (see Campbell, Vergés, et al., 2014; Egan et al., 2013 and references within). The direction and function of any mechanisms underpinning these associations should therefore be explored with more detailed research.

## 5 FUTURE DIRECTIONS AND CONCLUSIONS

The incorporation of genome‐wide host genetic data into microbiome studies is a novel approach that is only beginning to be applied to wild systems. Investigating host–microbiota interactions in marine species with this approach can also provide novel insights into the evolution and development of the holobiont because the environment, biology and ecology of marine systems are vastly different to terrestrial systems. Here, we demonstrate that this holistic approach can be used as a powerful tool to explain variation in microbial community data in marine systems. Our results are consistent with recent work spanning organisms as diverse as marine sponges (Easson et al., 2020), terrestrial plants (Bergelson et al., 2019; Peiffer et al., 2013) and mammals (Leamy et al., 2014; Rothschild et al., 2018) in showing that the local environment contributes a large amount of the compositional variation in the microbiome (Rothschild et al., 2018). Interestingly, we reveal that host traits, particularly genetics, play a significant role in shaping marine host‐associated microbial communities.

Given that bleaching, associated heat stress and herbivory represent some of the greatest threats to seaweed forests worldwide, investigations to determine if and how seaweed functioning and defence are linked to associated microbial communities are of critical importance. Further, potential manipulation of the environmental pool of microbes may have cascading effects if they alter nearby microbial communities in the water column (Chen & Parfrey, 2018; Lam & Harder, 2007) and on nearby hosts (e.g., corals Zaneveld et al., 2016). Future manipulative experiments, including breeding known genotypes and experimental outplanting or transplantation of hosts (Campbell et al., 2015; Quigley et al., 2018), may be used to tease out the effect of host genetics vs. geographical and environmental influences on microbial communities. These experiments could also assess how such changes in turn affect hosts (e.g., Burke et al., 2011), which should enable a better understanding of the biological effects of microbial taxa on hosts, and whether disruptions of host‐specific taxa have biological effects.

Management or manipulation of microbial functions and communities have become well established in bioremediation of terrestrial and aquatic ecosystems (e.g., Tyagi et al., 2011). Although the development of techniques to harness the beneficial effects of microbial interactions is still in its infancy in seaweed systems, changes in seaweed‐associated microbiota can have strong effects on the host (e.g., Egan et al., 2013). Rather than influences of single/specific taxa, there is often a microbial imbalance (e.g., Marzinelli et al., 2015; Qiu et al., 2019), or dysbiosis, as also occurs in the human gut and can lead to disease (e.g., Carding et al., 2015). In the same way as understanding dysbiosis and drivers of variation of the gut microbiota is revolutionizing biomedical science and human health management, understanding the drivers of variation in foundational seaweed‐associated microbiota (e.g., host genetics) can potentially transform how we manage the health of these ecosystems. Our work thus provides an important baseline to work towards similar restoration‐enhancing interventions, as well as to the development of rapid, cost‐effective assessment and monitoring tools under changing environmental conditions.

## AUTHOR CONTRIBUTIONS

All the authors conceived and designed the study; G.W. collected and analysed the data and led the writing of the manuscript. All the authors contributed critically to the drafts and gave final approval for publication.

## CONFLICT OF INTRESTS

The authors declare no conflict of interest.

## Supporting information

Supplementary Material

Supplementary Material

## Data Availability

Sampling locations, amplicon sequence data, morphological data and SNP genotype data are available from the Digital Dryad Repository (https://doi.org/10.5061/dryad.qz612jmd4).
