# Supplementary material for: Host genetics, phenotype and geography structure the microbiome of a foundational seaweed
Source: Mol Ecol. 2022 Feb 19;31(7):2189–206. doi: 10.1111/mec.16378 (PMC9540321; doi:10.1111/mec.16378)
Supplement: Supplementary file 1 — Supplementary Material [file MEC-31-2189-s002.docx]

**Host genetics, phenotype and geography structure the microbiome of a foundational seaweed**

**Wood,** **G**, Steinberg, PD, Campbell, AH, Vergés, A, Coleman, MA and Marzinelli, EM

**Online supplementary material**

Table S1. AMOVA between *Phyllospora* sites and regions (corresponding to rear, central and leading- edge populations).

| Source of variation |  | df | SS | Variance component* | % variation* |
| --- | --- | --- | --- | --- | --- |
| Among region |  | 2 | 401.65 | **2.40** | **14.32** |
| Among sites within region |  | 5 | 413.98 | **4.71** | **22.208** |
| Among samples within sites |  | 148 | 1570.02 | **10.61** | **63.46279** |
| Within all samples |  | 155 | 2385.66 | 16.72 | 100.00 |
|  |  |  |  |  |  |

*Significant values shown in bold.

**Table S2:** Adjusted p-values for pairwise comparisons of host genetic data between 8 sites.*

| Site ^a^ | AB | CR | ED | FO | MB | BI | PM |
| --- | --- | --- | --- | --- | --- | --- | --- |
| CR | **0.001** | **-** | **-** | **-** | **-** | **-** | **-** |
| ED | **0.001** | **0.001** | **-** | **-** | **-** | **-** | **-** |
| FO | **0.001** | **0.001** | **0.001** | **-** | **-** | **-** | **-** |
| MB | **0.001** | **0.001** | **0.001** | **0.001** | **-** | **-** | **-** |
| BI | **0.001** | **0.001** | **0.001** | **0.001** | **0.001** | **-** | **-** |
| PM | **0.001** | **0.001** | **0.001** | **0.001** | **0.001** | **0.001** | **-** |
| SO | **0.001** | **0.001** | **0.001** | **0.001** | **0.001** | **0.001** | **0.001** |

^*^ Values significant after FDR correction shown in bold.

^a^PM: Port Macquarie; FO: Forster; AB: Anna Bay; CR: Cronulla; MB: Malua Bay; ED: Eden; BI: Bicheno; SO: Southport.


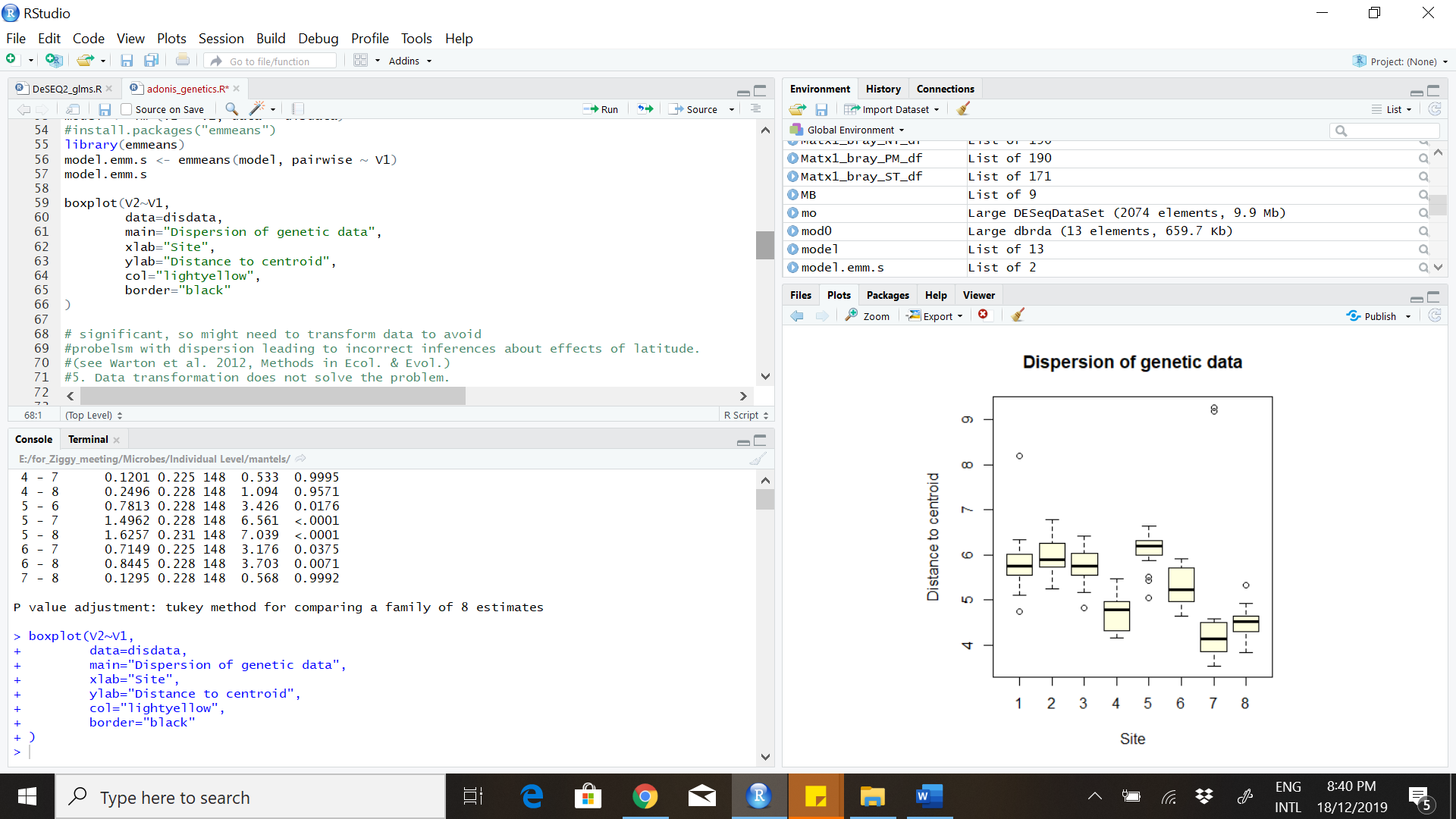


AB CR ED FO MB BI PM SO

Figure S1: Dispersion (i.e. variance) of *Phyllospora comosa* genetic data for 8 sites. PM: Port Macquarie; FO: Forster; AB: Anna Bay; CR: Cronulla; MB: Malua Bay; ED: Eden; BI: Bicheno; SO: Southport.

Table S3: Results of pairwise comparisons of dispersion in host genetic data between 8 sites. *

| Site ^a^ | AB | CR | ED | FO | MB | BI | PM |
| --- | --- | --- | --- | --- | --- | --- | --- |
| CR | 0.999 | **-** | **-** | **-** | **-** | **-** | **-** |
| ED | 1.000 | 0.987 | **-** | **-** | **-** | **-** | **-** |
| FO | **<0.001** | **<.001** | **0.002** | **-** | **-** | **-** | **-** |
| MB | 0.962 | 0.999 | 0.843 | **<.001** | **-** | **-** | **-** |
| BI | 0.278 | 0.083 | 0.469 | 0.149 | **0.018** | **-** | **-** |
| PM | **<.001** | **<.001** | **<.0001** | 0.999 | **<.001** | 0.038 | **-** |
| SO | **<.001** | **<.001** | **<.0001** | 0.957 | **<.001** | **0.007** | 0.999 |

^*^ Values significant after FDR correction shown in bold.

^a^PM: Port Macquarie; FO: Forster; AB: Anna Bay; CR: Cronulla; MB: Malua Bay; ED: Eden; BI: Bicheno; SO: Southport.

Table S4: Adjusted p-values for pairwise comparisons of host phenotype data between 8 sites.*

| Site ^a^ | AB | CR | ED | FO | MB | BI | PM |
| --- | --- | --- | --- | --- | --- | --- | --- |
| CR | **0.001** | **-** | **-** | **-** | **-** | **-** | **-** |
| ED | **0.001** | **0.001** | **-** | **-** | **-** | **-** | **-** |
| FO | **0.001** | **0.001** | **0.001** | **-** | **-** | **-** | **-** |
| MB | **0.001** | **0.001** | 0.249 | **0.013** | **-** | **-** | **-** |
| BI | **0.001** | **0.001** | **0.001** | **0.001** | **0.001** | **-** | **-** |
| PM | **0.001** | **0.001** | **0.001** | 0.124 | **0.010** | **0.001** | **-** |
| SO | **0.001** | **0.001** | **0.001** | **0.001** | **0.001** | **0.001** | **0.001** |

^*^ Values significant after FDR correction shown in bold.

^a^PM: Port Macquarie; FO: Forster; AB: Anna Bay; CR: Cronulla; MB: Malua Bay; ED: Eden; BI: Bicheno; SO: Southport.


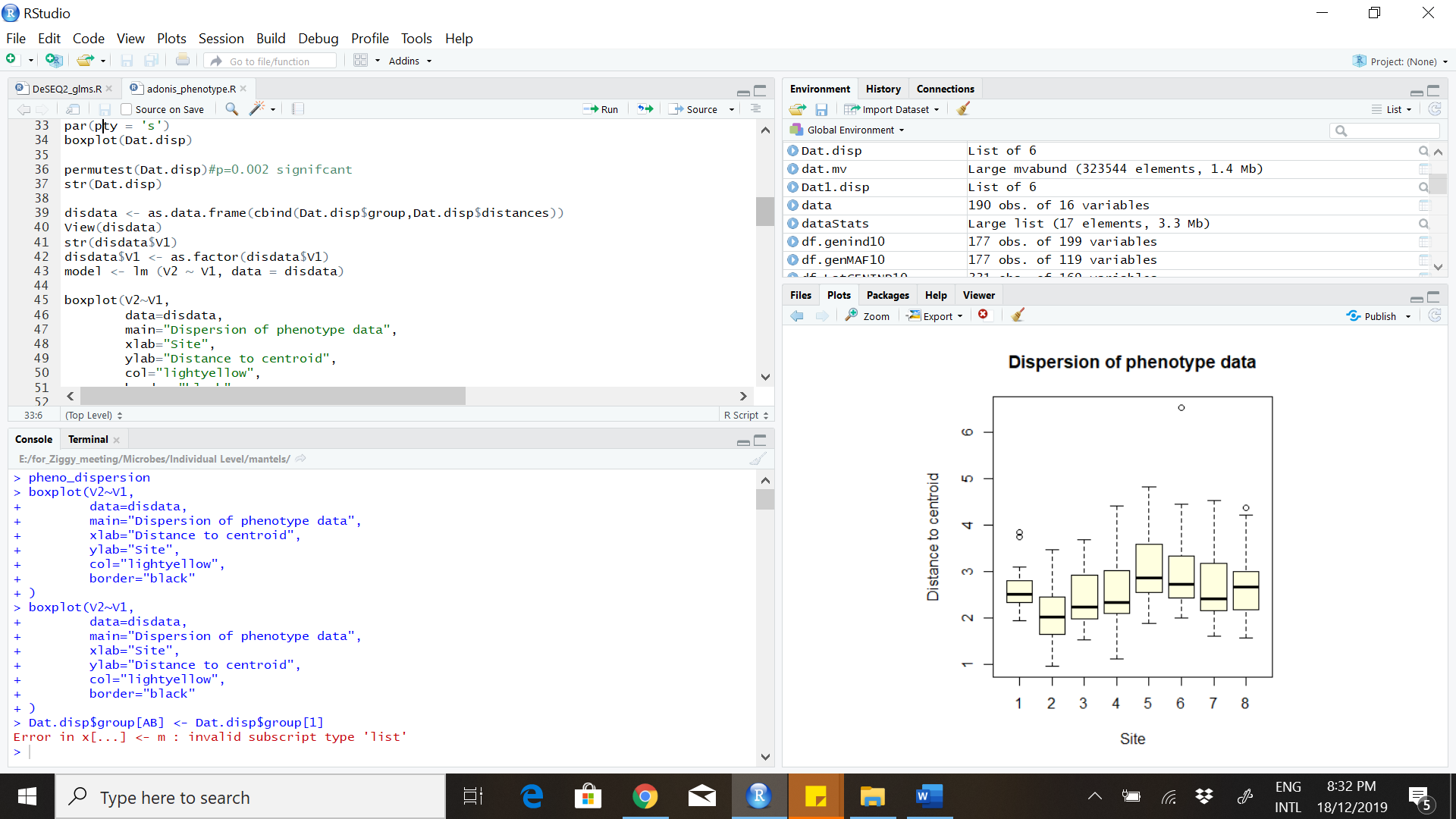


AB CR ED FO MB BI PM SO

Figure S2: Dispersion (i.e. variance) of *Phyllospora comosa* phenotype data for 8 sites. PM: Port Macquarie; FO: Forster; AB: Anna Bay; CR: Cronulla; MB: Malua Bay; ED: Eden; BI: Bicheno; SO: Southport.

Table S5: Results of pairwise comparisons of dispersion in host phenotype data between 8 sites. *

| Site ^a^ | AB | CR | ED | FO | MB | BI | PM |
| --- | --- | --- | --- | --- | --- | --- | --- |
| CR | 0.1587 | **-** | **-** | **-** | **-** | **-** | **-** |
| ED | 0.9474 | 0.8002 | **-** | **-** | **-** | **-** | **-** |
| FO | 1 | 0.3129 | 0.9941 | **-** | **-** | **-** | **-** |
| MB | 0.5496 | **0.0003** | 0.0511 | 0.3004 | **-** | **-** | **-** |
| BI | 0.8556 | **0.0021** | 0.1748 | 0.6243 | 0.9995 | **-** | **-** |
| PM | 1 | 0.1804 | 0.964 | 1 | 0.4713 | 0.7999 | **-** |
| SO | 1 | 0.1866 | 0.9634 | 1 | 0.4992 | 0.8189 | 1 |

^*^ Values significant after FDR correction shown in bold.

^a^PM: Port Macquarie; FO: Forster; AB: Anna Bay; CR: Cronulla; MB: Malua Bay; ED: Eden; BI: Bicheno; SO: Southport.


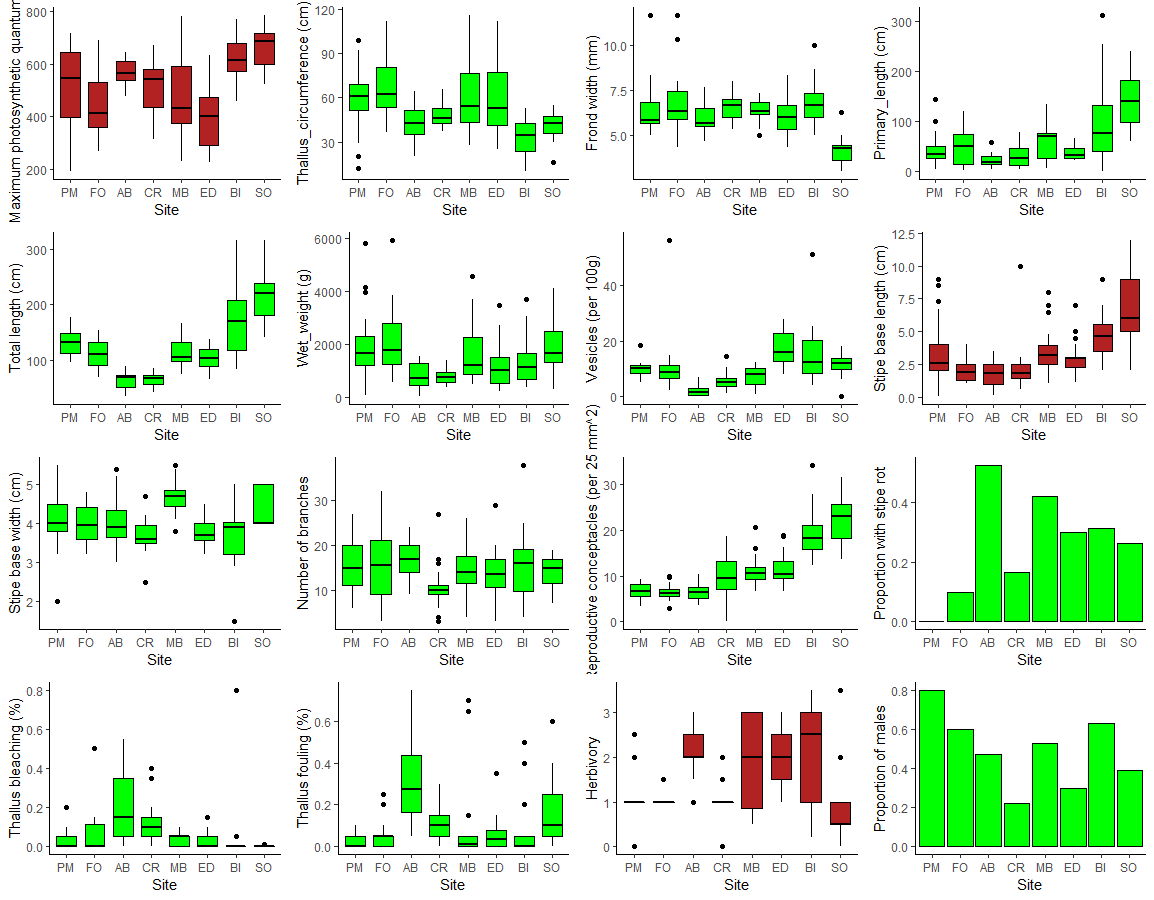


Figure S3: Plots of all phenotypic traits measured, ordered by site. Y-axes are labelled with the description of traits (not replicated here, for simplicity). Dark red plots indicate traits that were significantly associated with microbial community structure. Sites ordered north to south; PM: Port Macquarie; FO: Forster; AB: Anna Bay: CR: Cronulla; MB: Malua Bay; ED: Eden; BI: Bicheno; SO: Southport.

Table S6: Results of tests for differences between individual host phenotype data between 8 sites. *

| **Test** | **Trait** | **d.f** | **Stat** | **p** |  |
| --- | --- | --- | --- | --- | --- |
| *lm* | PAM | 7,142 | F = 10.62 | 1E-10 | * |
|  | Thallus circumference | 7,147 | F = 8.13 | 2E-08 | * |
|  | Frond width | 7,148 | F = 11.56 | 1E-11 | * |
|  | Log10 Primary length | 7,148 | F = 8.99 | 3.25E-09 | * |
|  | Log10 Total length | 7,148 | F = 54.73 | < 2.2e-16 | * |
|  | Wet weight | 7,148 | F = 5.909 | 5E-06 | * |
|  | Vesicles per 100g | 7,145 | F = 11.03 | 4E-11 | * |
|  | Stipe base length | 7,148 | F = 13.52 | 2E-13 | * |
|  | Stipe base width | 7,148 | F = 6.508 | 1E-06 | * |
|  | Thallus bleaching | 7,147 | F = 6.609 | 9E-07 | * |
|  | Thallus fouling | 7,146 | F = 8.655 | 7E-09 | * |
|  | Herbivory | 7,147 | F = 10.04 | 3E-10 | * |
|  | Reproductive conceptacles | 7,146 | F = 51.93 | < 2.2e-16 | * |
|  | Number of branches | 7,148 | F = 1.958 | 6E-02 |  |
| glm |  |  |  |  |  |
|  | **Trait** | **d.f** |  |  |  |
|  | Proportion with stipe rot | 7,143 | Dev =24.933 | 8E-04 | * |
|  | Proportion of males | 7,145 | Dev = 20.017 | 6E-03 | * |

^*^ Significant values in bold.

**Table S7:** Spearman correlation coefficient for all phenotypic traits measured.

|  | **a** | **b** | **c** | **d** | **e** | **f** | **g** | **h** | **i** | **j** | **k** | **l** | **m** | **n** | **o** | **p** |
| --- | --- | --- | --- | --- | --- | --- | --- | --- | --- | --- | --- | --- | --- | --- | --- | --- |
| Latitude^a^ |  |  |  |  |  |  |  |  |  |  |  |  |  |  |  |  |
| PAM^b^ | **0.210** |  |  |  |  |  |  |  |  |  |  |  |  |  |  |  |
| Circumference^c^ | **-0.371** | **-0.271** |  |  |  |  |  |  |  |  |  |  |  |  |  |  |
| Frond width^d^ | **-0.253** | **-0.237** | 0.115 |  |  |  |  |  |  |  |  |  |  |  |  |  |
| Primary length^e^ | **0.430** | 0.103 | -0.034 | -0.170 |  |  |  |  |  |  |  |  |  |  |  |  |
| Total length^f^ | **0.391** | **0.198** | 0.040 | **-0.243** | **0.688** |  |  |  |  |  |  |  |  |  |  |  |
| Wet weight^g^ | -0.044 | 0.011 | **0.627** | -0.093 | **0.376** | **0.557** |  |  |  |  |  |  |  |  |  |  |
| Vesicles per 100g^h^ | **0.356** | -0.110 | -0.039 | -0.146 | **0.383** | **0.520** | 0.147 |  |  |  |  |  |  |  |  |  |
| Stipe base length^i^ | **0.481** | **0.251** | **-0.243** | **-0.256** | **0.509** | **0.572** | **0.200** | **0.359** |  |  |  |  |  |  |  |  |
| Stipe base width^j^ | 0.044 | -0.011 | 0.081 | -0.061 | **0.210** | **0.199** | **0.187** | -0.134 | 0.022 |  |  |  |  |  |  |  |
| # branches^k^ | -0.046 | -0.006 | **0.242** | -0.054 | **0.417** | **0.222** | **0.299** | **0.246** | -0.014 | 0.071 |  |  |  |  |  |  |
| Conceptacles^l^ | **0.806** | 0.182 | **-0.312** | -0.122 | **0.414** | **0.432** | 0.002 | **0.370** | **0.524** | 0.084 | -0.066 |  |  |  |  |  |
| Stipe rot^m^ | **0.044** | 0.093 | -0.050 | -0.135 | -0.111 | -0.133 | -0.133 | -0.096 | -0.003 | 0.080 | -0.002 | 0.016 |  |  |  |  |
| Bleaching^n^ | **-0.301** | -0.041 | 0.008 | 0.142 | **-0.247** | **-0.447** | -0.168 | **-0.330** | **-0.365** | -0.053 | -0.058 | -0.368 | 0.152 |  |  |  |
| Fouling^o^ | 0.066 | 0.039 | -0.072 | -0.125 | -0.047 | -0.174 | -0.036 | **-0.301** | -0.102 | 0.101 | 0.050 | 0.000 | 0.159 | **0.365** |  |  |
| Herbivory^p^ | 0.111 | **0.323** | **-0.366** | 0.000 | **-0.227** | **-0.243** | **-0.498** | 0.067 | -0.115 | 0.036 | -0.024 | -0.002 | **0.320** | 0.125 | 0.031 |  |
| Sex | -0.168 | 0.060 | 0.173 | 0.040 | 0.256 | **0.215** | 0.163 | 0.016 | -0.074 | 0.166 | 0.240 | **-0.192** | 0.027 | -0.031 | -0.094 | 0.020 |

^*^ Values significant after FDR correction shown in bold.

**Table S8:** List of “core” microbial taxa, grouped by taxa. Each amplicon sequence variant (ASV) was present in all 156 individuals of the seaweed *Phyllospora comosa* sampled during a study spanning their entire latitudinal distribution. ASV IDs indicate rank order of relative abundance across all samples.

| ASV ID | Taxonomic details |  |  |  |  |
| --- | --- | --- | --- | --- | --- |
| ASV10 | Proteobacteria | Gammaproteobacteria | Arenicellales | Arenicellaceae | Arenicella |
| ASV15 | Proteobacteria | Gammaproteobacteria | Arenicellales | Arenicellaceae | Arenicella |
| ASV31 | Proteobacteria | Gammaproteobacteria | Arenicellales | Arenicellaceae | Arenicella |
| ASV7 | Proteobacteria | Gammaproteobacteria | Thiohalorhabdales | Thiohalorhabdaceae | Granulosicoccus |
| ASV9 | Proteobacteria | Gammaproteobacteria | Thiohalorhabdales | Thiohalorhabdaceae | Granulosicoccus |
| ASV141 | Planctomycetes | Planctomycetacia | Pirellulales | Pirellulaceae | Blastopirellula |
| ASV182 | Planctomycetes | Planctomycetacia | Pirellulales | Pirellulaceae | Blastopirellula |
| ASV3 | Planctomycetes | Planctomycetacia | Pirellulales | Pirellulaceae | Blastopirellula |
| ASV4 | Planctomycetes | Planctomycetacia | Pirellulales | Pirellulaceae | Blastopirellula |
| ASV43 | Planctomycetes | Planctomycetacia | Pirellulales | Pirellulaceae | Blastopirellula |
| ASV8 | Planctomycetes | Planctomycetacia | Pirellulales | Pirellulaceae | Blastopirellula |
| ASV155 | Proteobacteria | Alphaproteobacteria | Caulobacterales | Hyphomonadaceae | Hellea |
| ASV351 | Proteobacteria | Alphaproteobacteria | Caulobacterales | Hyphomonadaceae | Hellea |
| ASV5 | Proteobacteria | Alphaproteobacteria | Caulobacterales | Hyphomonadaceae | Hellea |
| ASV57 | Proteobacteria | Alphaproteobacteria | Caulobacterales | Hyphomonadaceae | Hellea |
| ASV529 | Proteobacteria | Alphaproteobacteria | Caulobacterales | Hyphomonadaceae | Litorimonas |
| ASV6 | Proteobacteria | Alphaproteobacteria | Caulobacterales | Hyphomonadaceae | Litorimonas |
| ASV1 | Proteobacteria | Alphaproteobacteria | Caulobacterales | Hyphomonadaceae | uncultured |
| ASV68 | Proteobacteria | Alphaproteobacteria | Caulobacterales | Hyphomonadaceae | uncultured |
| ASV23 | Proteobacteria | Alphaproteobacteria | Rhodobacterales | Rhodobacteraceae | uncultured |
| ASV13 | Verrucomicrobia | Verrucomicrobiae | Verrucomicrobiales | Rubritaleaceae | Rubritalea |
| ASV44 | Verrucomicrobia | Verrucomicrobiae | Verrucomicrobiales | Rubritaleaceae | Rubritalea |
| ASV2 | Cyanobacteria | Oxyphotobacteria |  |  |  |

Tables S9: a-c (i-iii): Adjusted p-values for pairwise comparisons of (a) overall, (b) variable and (c) core microbial community data between 8 sites, based on Bray-Curtis dissimilarity matrix. *

(a)

| Site ^a^ | AB | CR | ED | FO | MB | BI | PM |
| --- | --- | --- | --- | --- | --- | --- | --- |
| CR | **0.001** | **-** | **-** | **-** | **-** | **-** | **-** |
| ED | **0.001** | **0.001** | **-** | **-** | **-** | **-** | **-** |
| FO | **0.001** | **0.001** | **0.001** | **-** | **-** | **-** | **-** |
| MB | **0.001** | **0.001** | **0.001** | **0.001** | **-** | **-** | **-** |
| BI | **0.001** | **0.001** | **0.001** | **0.001** | **0.001** | **-** | **-** |
| PM | **0.001** | **0.001** | **0.001** | **0.001** | **0.001** | **0.001** | **-** |
| SO | **0.001** | **0.001** | **0.001** | **0.001** | **0.001** | **0.001** | **0.001** |

(b)

| Site ^a^ | AB | CR | ED | FO | MB | BI | PM |
| --- | --- | --- | --- | --- | --- | --- | --- |
| CR | **0.001** | **-** | **-** | **-** | **-** | **-** | **-** |
| ED | **0.001** | **0.001** | **-** | **-** | **-** | **-** | **-** |
| FO | **0.001** | **0.001** | **0.001** | **-** | **-** | **-** | **-** |
| MB | **0.001** | **0.001** | **0.001** | **0.001** | **-** | **-** | **-** |
| BI | **0.001** | **0.001** | **0.001** | **0.001** | **0.001** | **-** | **-** |
| PM | **0.001** | **0.001** | **0.001** | **0.001** | **0.001** | **0.001** | **-** |
| SO | **0.001** | **0.001** | **0.001** | **0.001** | **0.001** | **0.001** | **0.001** |

(c)

| Site ^a^ | AB | CR | ED | FO | MB | BI | PM |
| --- | --- | --- | --- | --- | --- | --- | --- |
| CR | 0.262 | **-** | **-** | **-** | **-** | **-** | **-** |
| ED | **0.001** | **0.001** | **-** | **-** | **-** | **-** | **-** |
| FO | **0.030** | 0.050 | **0.001** | **-** | **-** | **-** | **-** |
| MB | **0.006** | **0.003** | **0.001** | **0.018** | **-** | **-** | **-** |
| BI | **0.001** | **0.001** | **0.001** | **0.001** | **0.001** | **-** | **-** |
| PM | **0.001** | **0.001** | **0.001** | **0.001** | **0.001** | **0.001** | **-** |
| SO | **0.001** | **0.001** | **0.004** | **0.001** | **0.001** | **0.001** | **0.001** |

^*^ Values significant after FDR correction shown in bold.

^a^PM: Port Macquarie; FO: Forster; AB: Anna Bay; CR: Cronulla; MB: Malua Bay; ED: Eden; BI: Bicheno; SO: Southport.


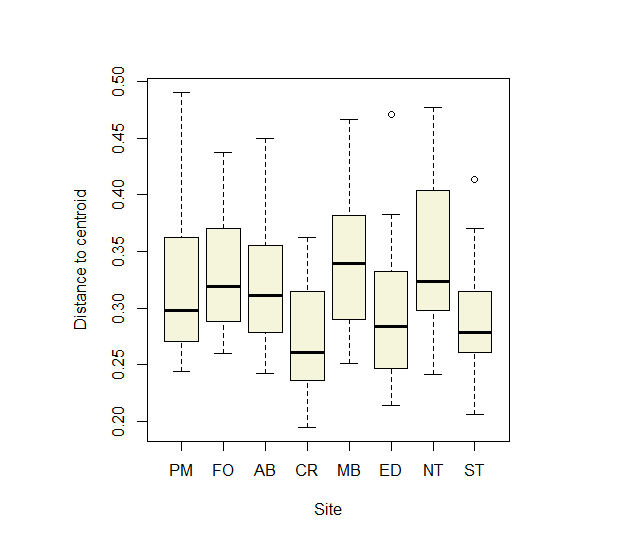
(a)


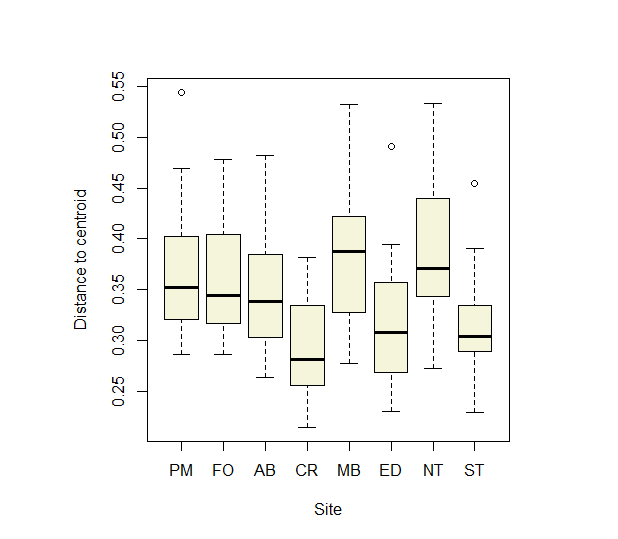
(b)


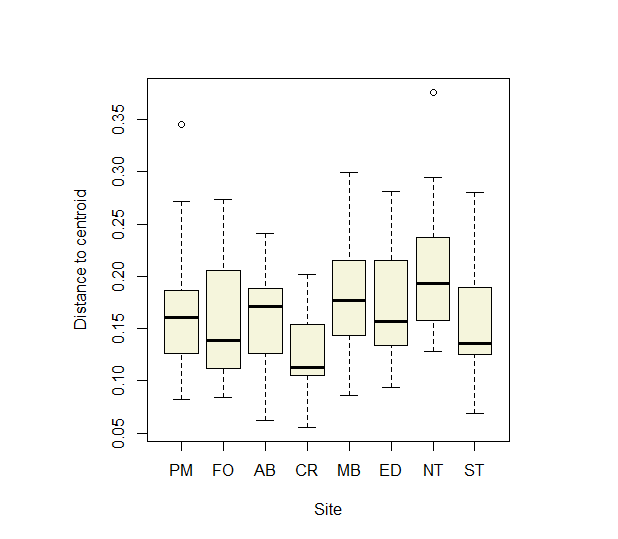
(c)

Figures S4: a-c: Dispersion (i.e. variance) of *Phyllospora comosa* (a) overall, (b) variable and (c) core microbial community data for 8 sites, based on Bray-Curtis dissimilarity matrix. PM: Port Macquarie; FO: Forster; AB: Anna Bay: CR: Cronulla; MB: Malua Bay; ED: Eden; BI: Bicheno; SO: Southport.

Tables S10: a-c: Results of pairwise comparisons of dispersion in (a) overall, (b) variable and (c) core microbial community data between 8 sites, based on Bray-Curtis dissimilarity matrix based on overall microbial data . *

| contrast | estimate | SE | df | t.ratio | p.value |
| --- | --- | --- | --- | --- | --- |
| PM - FO | -0.006774 | 0.0189 | 148 | -0.358 | 1.0000 |
| PM - AB | 0.006996 | 0.0191 | 148 | 0.365 | 1.0000 |
| PM - CR | 0.051634 | 0.0191 | 148 | 2.697 | 0.1316 |
| PM - MB | -0.020634 | 0.0191 | 148 | -1.078 | 0.9605 |
| PM - ED | 0.028424 | 0.0189 | 148 | 1.504 | 0.8041 |
| PM - BI | -0.020983 | 0.0189 | 148 | -1.111 | 0.9536 |
| PM - SO | 0.034373 | 0.0191 | 148 | 1.796 | 0.6240 |
| FO - AB | 0.013770 | 0.0191 | 148 | 0.719 | 0.9963 |
| FO - CR | 0.058408 | 0.0191 | 148 | 3.051 | 0.0534 |
| FO - MB | -0.013860 | 0.0191 | 148 | -0.724 | 0.9962 |
| FO - ED | 0.035198 | 0.0189 | 148 | 1.863 | 0.5785 |
| FO - BI | -0.014209 | 0.0189 | 148 | -0.752 | 0.9951 |
| FO - SO | 0.041146 | 0.0191 | 148 | 2.150 | 0.3886 |
| AB - CR | 0.044638 | 0.0194 | 148 | 2.303 | 0.2992 |
| AB - MB | -0.027630 | 0.0194 | 148 | -1.425 | 0.8442 |
| AB - ED | 0.021428 | 0.0191 | 148 | 1.119 | 0.9517 |
| AB - BI | -0.027979 | 0.0191 | 148 | -1.462 | 0.8263 |
| AB - SO | 0.027376 | 0.0194 | 148 | 1.412 | 0.8504 |
| **CR - MB** | **-0.072268** | **0.0194** | **148** | **-3.728** | **0.0065** |
| CR - ED | -0.023210 | 0.0191 | 148 | -1.213 | 0.9271 |
| **CR - BI** | **-0.072617** | **0.0191** | **148** | **-3.794** | **0.0052** |
| CR - SO | -0.017262 | 0.0194 | 148 | -0.890 | 0.9865 |
| MB - ED | 0.049058 | 0.0191 | 148 | 2.563 | 0.1782 |
| MB - BI | -0.000349 | 0.0191 | 148 | -0.018 | 1.0000 |
| MB - SO | 0.055007 | 0.0194 | 148 | 2.838 | 0.0937 |
| ED - BI | -0.049407 | 0.0189 | 148 | -2.615 | 0.1589 |
| ED - SO | 0.005949 | 0.0191 | 148 | 0.311 | 1.0000 |
| BI - SO | 0.055356 | 0.0191 | 148 | 2.892 | 0.0816 |

(a)

^*^ Values significant after FDR correction shown in bold.

^a^PM: Port Macquarie; FO: Forster; AB: Anna Bay; CR: Cronulla; MB: Malua Bay; ED: Eden; BI: Bicheno; SO: Southport.

^*^ Values significant after FDR correction shown in bold.

(b)

| contrast | estimate | SE | df | t.ratio | p.value |
| --- | --- | --- | --- | --- | --- |
| PM - FO | 0.010251 | 0.0176 | 148 | 0.583 | 0.9990 |
| PM - AB | 0.011647 | 0.0178 | 148 | 0.654 | 0.9980 |
| PM - CR | 0.045368 | 0.0178 | 148 | 2.548 | 0.1838 |
| PM - MB | -0.007674 | 0.0178 | 148 | -0.431 | 0.9999 |
| PM - ED | -0.001791 | 0.0176 | 148 | -0.102 | 1.0000 |
| PM - BI | -0.031916 | 0.0176 | 148 | -1.816 | 0.6101 |
| PM - SO | 0.012611 | 0.0178 | 148 | 0.708 | 0.9966 |
| FO - AB | 0.001396 | 0.0178 | 148 | 0.078 | 1.0000 |
| FO - CR | 0.035117 | 0.0178 | 148 | 1.973 | 0.5038 |
| FO - MB | -0.017925 | 0.0178 | 148 | -1.007 | 0.9728 |
| FO - ED | -0.012042 | 0.0176 | 148 | -0.685 | 0.9973 |
| FO - BI | -0.042167 | 0.0176 | 148 | -2.400 | 0.2493 |
| FO - SO | 0.002360 | 0.0178 | 148 | 0.133 | 1.0000 |
| AB - CR | 0.033720 | 0.0180 | 148 | 1.870 | 0.5733 |
| AB - MB | -0.019321 | 0.0180 | 148 | -1.072 | 0.9617 |
| AB - ED | -0.013439 | 0.0178 | 148 | -0.755 | 0.9950 |
| AB - BI | -0.043563 | 0.0178 | 148 | -2.447 | 0.2270 |
| AB - SO | 0.000964 | 0.0180 | 148 | 0.053 | 1.0000 |
| CR - MB | -0.053042 | 0.0180 | 148 | -2.942 | 0.0716 |
| CR - ED | -0.047159 | 0.0178 | 148 | -2.649 | 0.1471 |
| **CR - BI** | **-0.077284** | **0.0178** | **148** | **-4.341** | **0.0007** |
| CR - SO | -0.032756 | 0.0180 | 148 | -1.817 | 0.6097 |
| MB - ED | 0.005883 | 0.0178 | 148 | 0.330 | 1.0000 |
| MB - BI | -0.024242 | 0.0178 | 148 | -1.362 | 0.8729 |
| MB - SO | 0.020285 | 0.0180 | 148 | 1.125 | 0.9503 |
| ED - BI | -0.030124 | 0.0176 | 148 | -1.714 | 0.6780 |
| ED - SO | 0.014403 | 0.0178 | 148 | 0.809 | 0.9924 |
| BI - SO | 0.044527 | 0.0178 | 148 | 2.501 | 0.2031 |

^*^

^*^ Values significant after FDR correction shown in bold.

^a^PM: Port Macquarie; FO: Forster; AB: Anna Bay; CR: Cronulla; MB: Malua Bay; ED: Eden; BI: Bicheno; SO: Southport.

(c)

| contrast | estimate | SE | df | t.ratio | p.value |
| --- | --- | --- | --- | --- | --- |
| PM - FO | 0.010251 | 0.0176 | 148 | 0.583 | 0.9990 |
| PM - AB | 0.011647 | 0.0178 | 148 | 0.654 | 0.9980 |
| PM - CR | 0.045368 | 0.0178 | 148 | 2.548 | 0.1838 |
| PM - MB | -0.007674 | 0.0178 | 148 | -0.431 | 0.9999 |
| PM - ED | -0.001791 | 0.0176 | 148 | -0.102 | 1.0000 |
| PM - BI | -0.031916 | 0.0176 | 148 | -1.816 | 0.6101 |
| PM - SO | 0.012611 | 0.0178 | 148 | 0.708 | 0.9966 |
| FO - AB | 0.001396 | 0.0178 | 148 | 0.078 | 1.0000 |
| FO - CR | 0.035117 | 0.0178 | 148 | 1.973 | 0.5038 |
| FO - MB | -0.017925 | 0.0178 | 148 | -1.007 | 0.9728 |
| FO - ED | -0.012042 | 0.0176 | 148 | -0.685 | 0.9973 |
| FO - BI | -0.042167 | 0.0176 | 148 | -2.400 | 0.2493 |
| FO - SO | 0.002360 | 0.0178 | 148 | 0.133 | 1.0000 |
| AB - CR | 0.033720 | 0.0180 | 148 | 1.870 | 0.5733 |
| AB - MB | -0.019321 | 0.0180 | 148 | -1.072 | 0.9617 |
| AB - ED | -0.013439 | 0.0178 | 148 | -0.755 | 0.9950 |
| AB - BI | -0.043563 | 0.0178 | 148 | -2.447 | 0.2270 |
| AB - SO | 0.000964 | 0.0180 | 148 | 0.053 | 1.0000 |
| CR - MB | -0.053042 | 0.0180 | 148 | -2.942 | 0.0716 |
| CR - ED | -0.047159 | 0.0178 | 148 | -2.649 | 0.1471 |
| **CR - BI** | **-0.077284** | **0.0178** | **148** | **-4.341** | **0.0007** |
| CR - SO | -0.032756 | 0.0180 | 148 | -1.817 | 0.6097 |
| MB - ED | 0.005883 | 0.0178 | 148 | 0.330 | 1.0000 |
| MB - BI | -0.024242 | 0.0178 | 148 | -1.362 | 0.8729 |
| MB - SO | 0.020285 | 0.0180 | 148 | 1.125 | 0.9503 |
| ED - BI | -0.030124 | 0.0176 | 148 | -1.714 | 0.6780 |
| ED - SO | 0.014403 | 0.0178 | 148 | 0.809 | 0.9924 |
| BI - SO | 0.044527 | 0.0178 | 148 | 2.501 | 0.2031 |

^*^ Values significant after FDR correction shown in bold.

^a^PM: Port Macquarie; FO: Forster; AB: Anna Bay; CR: Cronulla; MB: Malua Bay; ED: Eden; BI: Bicheno; SO: Southport.

Figure S5: Relationship between site-level host genetic diversity (H_E_) and a) species richness and b) Simpson diversity of microbial communities associated with the seaweed *Phyllospora comosa*’s surface. Data collected at eight sites.


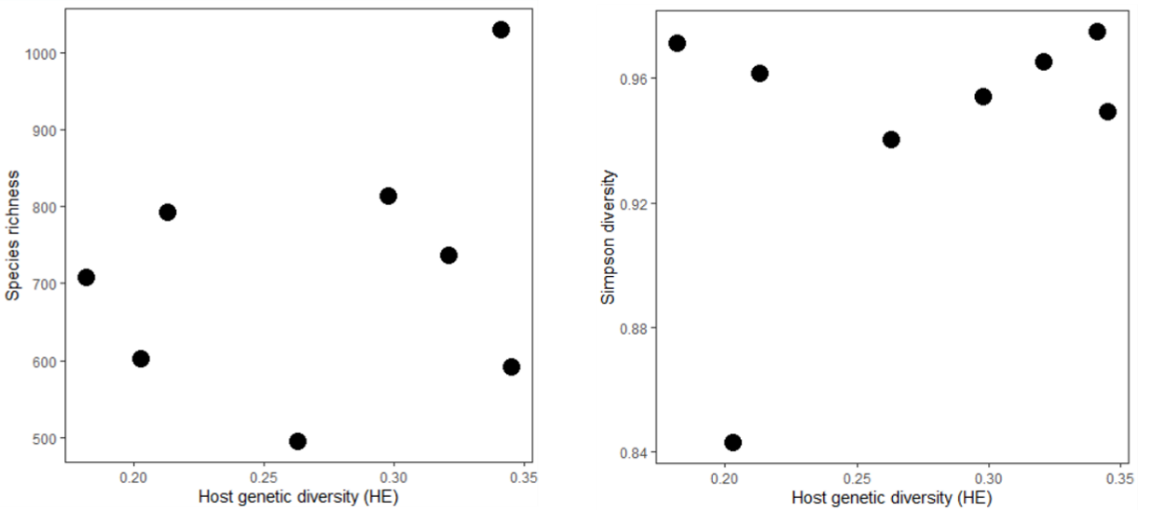


Host genetic diversity (H_E_)

Host genetic diversity (H_E_)

Species richness

Simpson diversity

Table S11 a-c: Mantel tests results describing relationship between (i) *Phyllospora comosa* genetic distance (Euclidean) and (ii) phenotypic distance (Euclidean) on surface-associated microbial community dissimilarities calculated using Bray-Curtis on square-root transformed relative abundances for (a) the overall microbial community dataset, (b) core microbial taxa and (c) variable microbial taxa only.*

a)

| Site ^a^ | Genetics | | | | Phenotype | |
| --- | --- | --- | --- | --- | --- | --- |
|  | Overall microbiome | | | Overall microbiome | | |
|  | Mantel r | p | Mantel r | | | p |
| PM | -0.166 | 0.775 | -0.1767 | | | 0.87 |
| FO | 0.089 | 0.194 | 0.233 | | | 0.056 |
| AB | **0.468** | **0.002** | 0.023 | | | 0.4 |
| CR | -0.004 | 0.545 | 0.094 | | | 0.283 |
| MB | 0.014 | 0.498 | -0.061 | | | 0.664 |
| ED | 0.125 | 0.089 | 0.1721 | | | 0.078 |
| BI | -0.055 | 0.703 | **0.221** | | | **0.045** |
| SO | -0.2044 | 0.979 | -0.0156 | | | 0.536 |

b)

| Site ^a^ | Genetics | | | | Phenotype | |
| --- | --- | --- | --- | --- | --- | --- |
|  | Core microbiome | | | Core microbiome | | |
|  | Mantel r | p | Mantel r | | | p |
| PM | -0.059 | 0.528 | -0.076 | | | 0.693 |
| FO | 0.044 | 0.348 | **0.328** | | | **0.015** |
| AB | **0.370** | **0.004** | 0.002 | | | 0.483 |
| CR | **0.350** | **0.038** | 0.436 | | | 0.025 |
| MB | 0.047 | 0.392 | -0.012 | | | 0.541 |
| ED | 0.151 | 0.059 | **0.210** | | | **0.043** |
| BI | -0.103 | 0.815 | 0.193 | | | 0.081 |
| SO | -0.137 | 0.904 | -0.088 | | | 0.768 |

c)

| Site ^a^ | Genetics | | | | Phenotype | |
| --- | --- | --- | --- | --- | --- | --- |
|  | Variable microbiome | | | Variable microbiome | | |
|  | Mantel r | p | Mantel r | | | p |
| PM | -0.126 | 0.732 | -0.150 | | | 0.827 |
| FO | 0.115 | 0.109 | 0.235 | | | 0.052 |
| AB | **0.486** | **0.001** | 0.046 | | | 0.315 |
| CR | -0.017 | 0.573 | 0.060 | | | 0.378 |
| MB | 0.098 | 0.216 | -0.069 | | | 0.674 |
| ED | 0.115 | 0.120 | 0.154 | | | 0.115 |
| BI | -0.052 | 0.692 | **0.232** | | | **0.022** |
| SO | -0.198 | 0.971 | 0.009 | | | 0.453 |

^*^ Values significant after FDR correction shown in bold.

^a^PM: Port Macquarie; FO: Forster; AB: Anna Bay: BB: Bateau Bay: TE: Terrigal: PB: Palm Beach; CR: Cronulla; SP: Shark Park; SH: Shellharbour; MB: Malua Bay; ED: Eden; BI: Bicheno; SO: Southport.

Table S12: Microbial taxa significantly related to geography, host phenotypic and genetic traits shown to influence overall microbial community, as identified using DEseq2.

| **Variable** | **Number of associated ASVs** |
| --- | --- |
| Site | 1199 |
| Maximum quantum yield | 17 |
| Herbivory | 13 |
| Stipe base length | 31 |
| Locus 28125_un_3937436 | 36 |
| Locus 40713_un_5699768 | 13 |
| Locus 52118_un_7296457 | 50 |


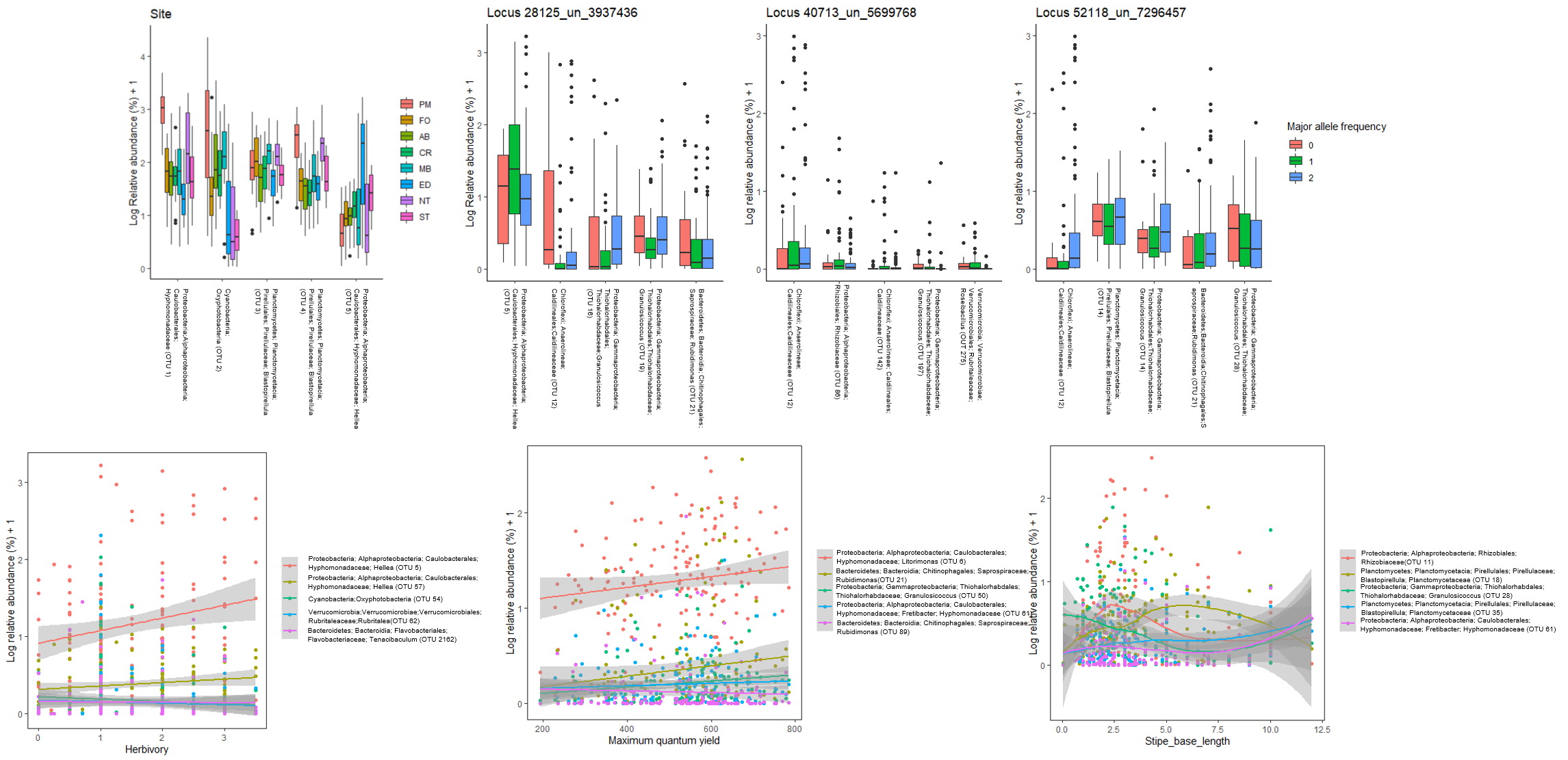


BI

SO

(S6a)


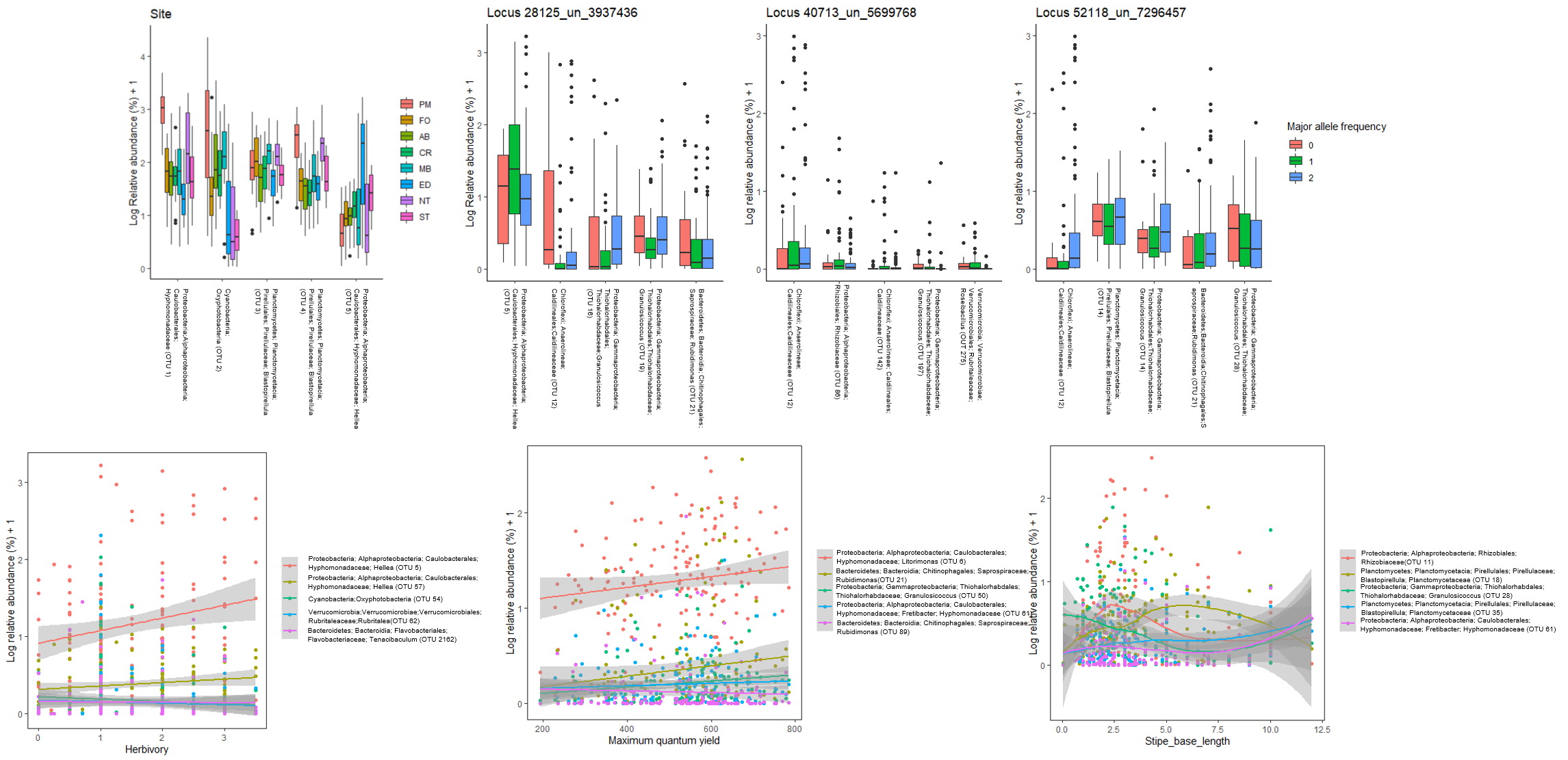


(S6b)

(S6c)


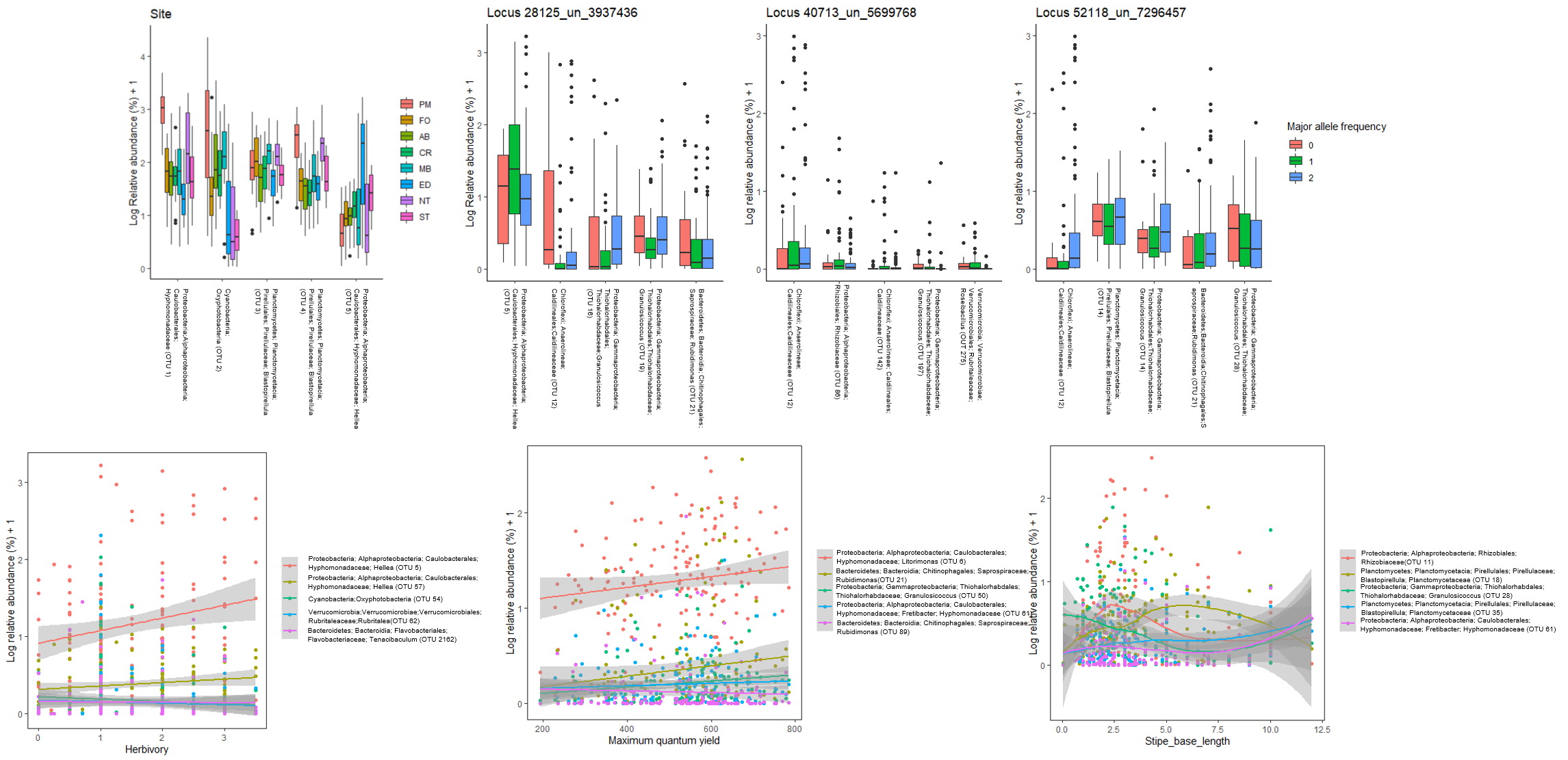

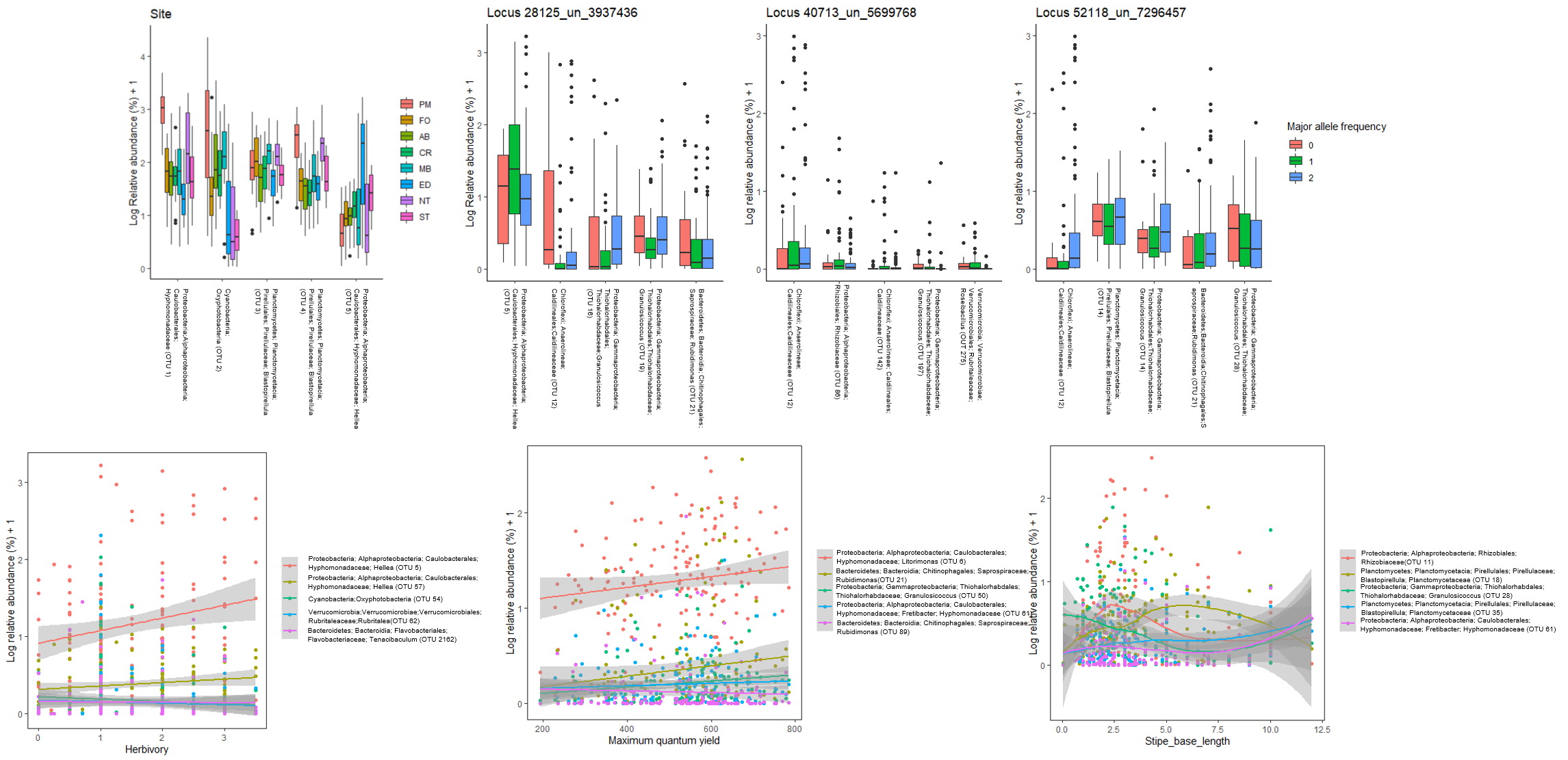

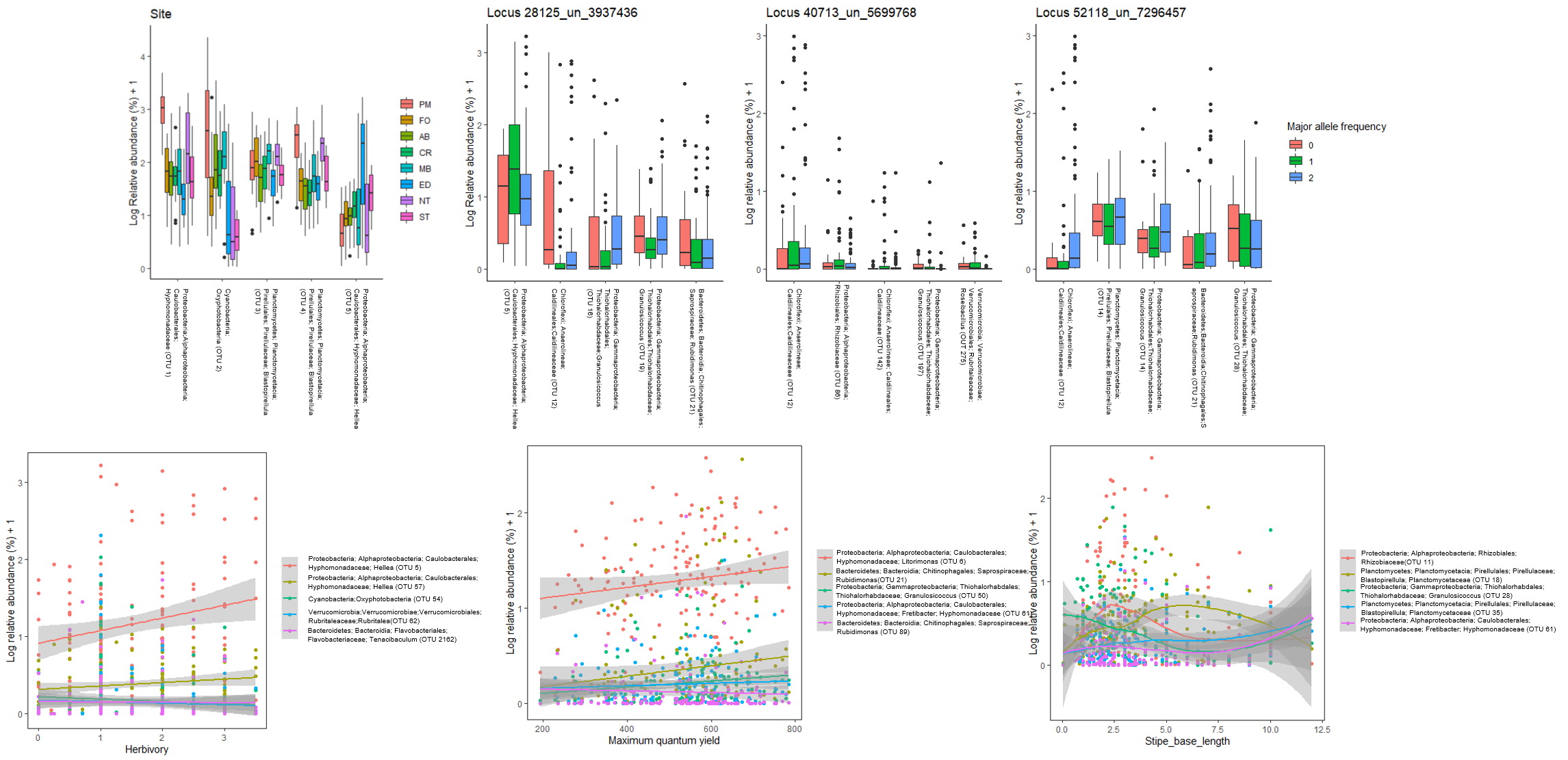


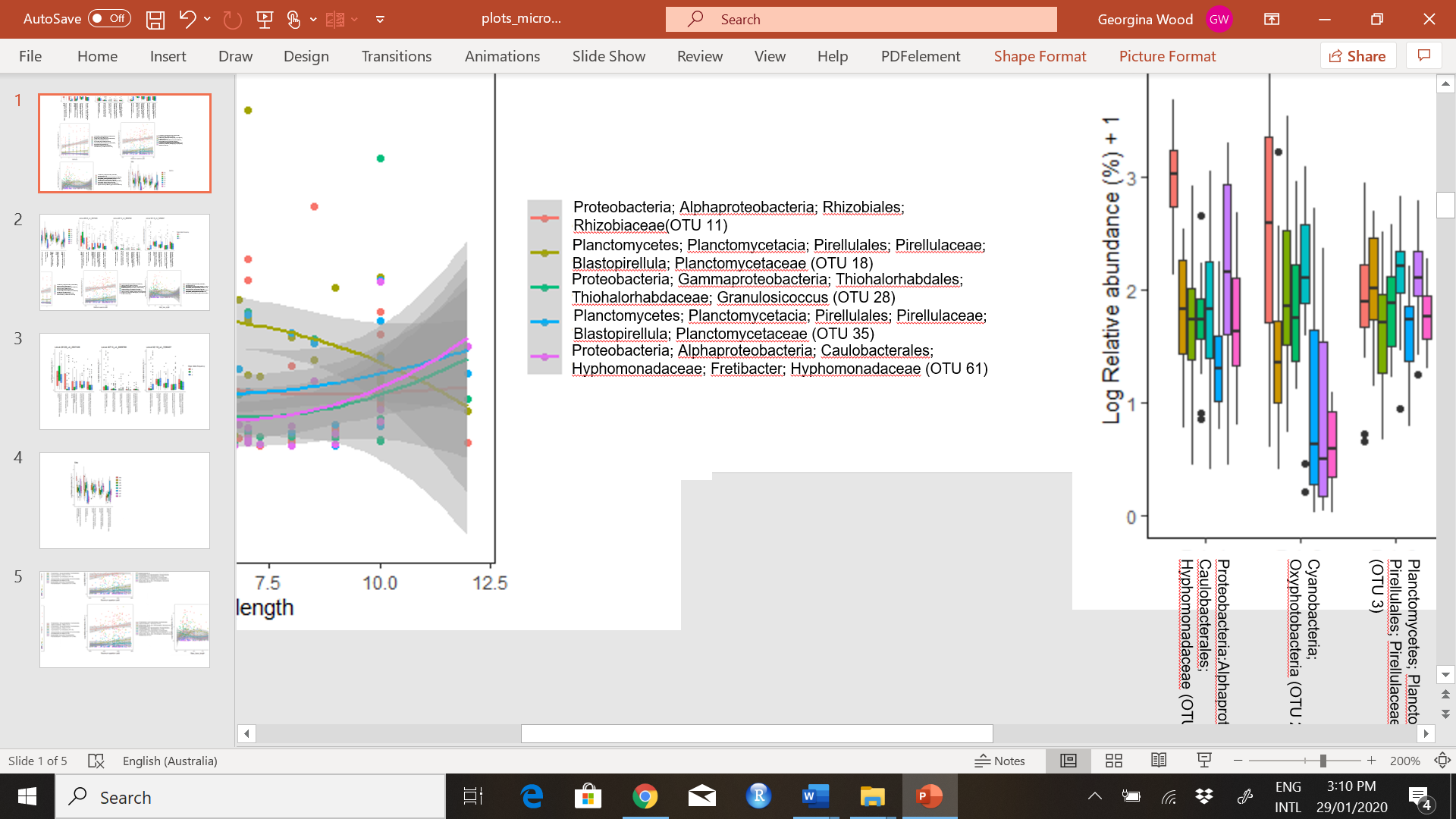

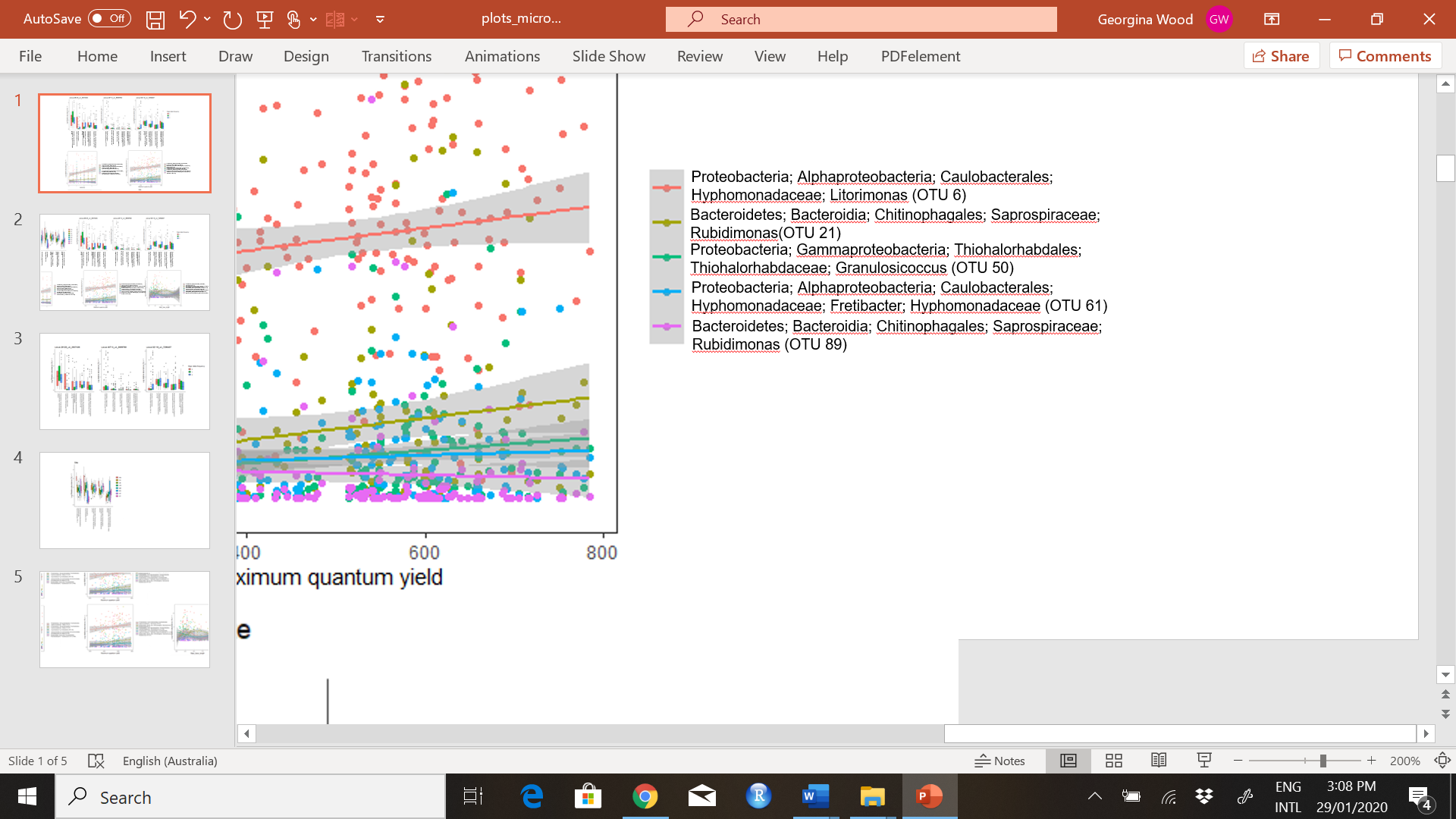

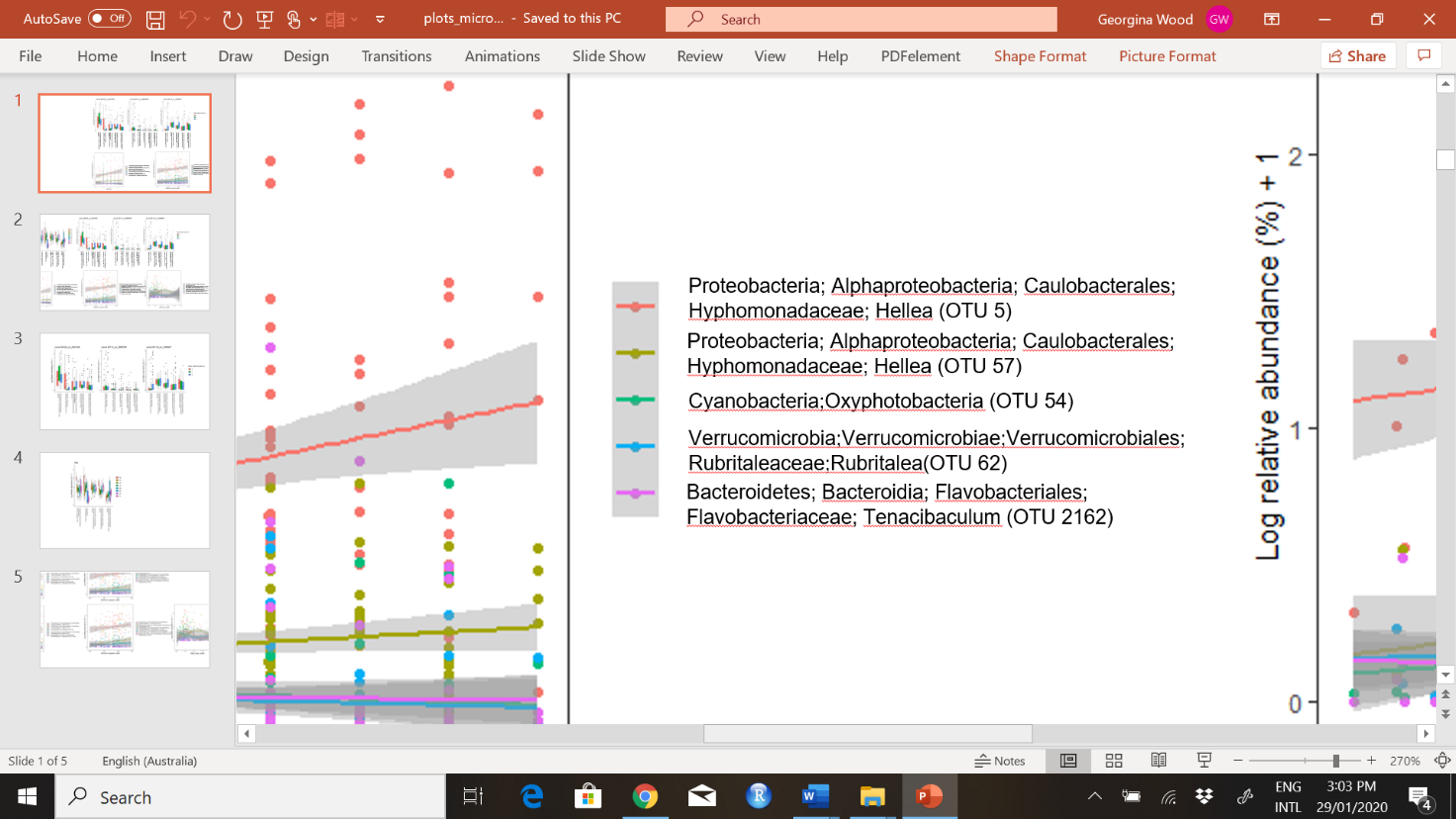


Figure S6 a-c: Relationship between (a) geography, (b) host genetics and (c) phenotype on *Phyllospora comosa’s* overall microbial communities. Taxa shown are the five most abundant ASVs that were significantly associated with each respective variable of interest. 0, 1 and 2 refer to the number of major alleles in each genotype; e.g. a genotype of 2 refers to a genotype with both dominant alleles present. Sites ordered north to south; PM: Port Macquarie; FO: Forster; AB: Anna Bay: CR: Cronulla; MB: Malua Bay; ED: Eden; BI: Bicheno; SO: Southport.

End of document.
